# Immunological properties of oxygen-transport proteins: hemoglobin, hemocyanin and hemerythrin

**DOI:** 10.1007/s00018-016-2326-7

**Published:** 2016-08-12

**Authors:** Christopher J. Coates, Heinz Decker

**Affiliations:** 1 Department of Biosciences, College of Science, Swansea University, Singleton Park, Swansea, Wales SA2 8PP UK; 2 Institut für Molekulare Biophysik, Johannes Gutenberg-Universität Mainz, Jakob Welder-Weg 28, 55128 Mainz, Germany

**Keywords:** Antimicrobial peptides, Innate immunity, Myoglobin, Phenoloxidase, Erythrocytes, Enzyme promiscuity, Metabolism, Redox

## Abstract

It is now well documented that peptides with enhanced or alternative functionality (termed cryptides) can be liberated from larger, and sometimes inactive, proteins. A primary example of this phenomenon is the oxygen-transport protein hemoglobin. Aside from respiration, hemoglobin and hemoglobin-derived peptides have been associated with immune modulation, hematopoiesis, signal transduction and microbicidal activities in metazoans. Likewise, the functional equivalents to hemoglobin in invertebrates, namely hemocyanin and hemerythrin, act as potent immune effectors under certain physiological conditions. The purpose of this review is to evaluate the true extent of oxygen-transport protein dynamics in innate immunity, and to impress upon the reader the multi-functionality of these ancient proteins on the basis of their structures. In this context, erythrocyte–pathogen antibiosis and the immune competences of various erythroid cells are compared across diverse taxa.

**Electronic supplementary material:**

The online version of this article (doi:10.1007/s00018-016-2326-7) contains supplementary material, which is available to authorized users.

## Introduction

Maintaining immunity-related proteostasis in metazoans is crucial to survival and recovery from biotic (pathogenic) and abiotic (environmental) traumas. Enlisting oxygen-transport proteins (OTPs: hemoglobin and hemocyanin) to directly combat microbes as well as to supply the O_2_ necessary to fuel the costly immuno-metabolome is both resourceful and economical. The concentration of oxygen within wounds determines a hosts’ ability to heal and to resist microbial colonisation [1, 2]. Hypoxia and inadequate oxygen tension within tissues can compromise immune cell functionality, e.g. restricting neutrophil respiratory burst [3].

With representatives in almost every known taxon, hemoglobin (Hb), hemocyanin (Hc) and hemerythrin (Hr) are metallated proteins responsible primarily for the sensing, transport and/or storage of O_2_ [4]. Hb and Hr are located within the corpuscles (erythrocytes) of vertebrate blood and specialised immune cells (hemerythrocytes) of invertebrate coelomic fluid^1^, respectively [5]. Conversely, Hcs are freely dissolved within the hemolymph plasma of some molluscs and arthropods. Although present in fewer species, it must be noted that two other forms of heme-based OTPs exist, namely chlorocruorin and erythrocruorin from annelids (often referred to as giant extracellular hemoglobins: GE-Hbs) [6–8].

Reports of Hb’s involvement in anti-infective defences have existed since the late 1950s [9], yet only in the last two decades has it become evident that Hb, Hc, and to a lesser extent Hr, contribute to various innate immune mechanisms. Generation of bioactive peptides (cryptides), enzyme promiscuity and pro-inflammatory signalling are but some of the many functions attributed to OTPs, which are expanded upon herein.

## The oxygen-transport proteins

### Hemoglobins

The presence and diversity of hemoglobins (Hbs) have been confirmed in metazoans, prokaryotes, fungi and flora, with a notable absence in icefish. A common ancestor for globin-related respiratory proteins existed over 1.5 billion years ago [reviewed by 10]. In excess of 250 million Hb molecules are packaged tightly within each human erythrocyte, guaranteeing their stability against proteolysis, a low colloid osmotic pressure, and preventing loss by filtration in the kidneys. Hb concentration within healthy adults ranges from 120 to 160 mg mL^−1^ [11].

**Fig. 1:**
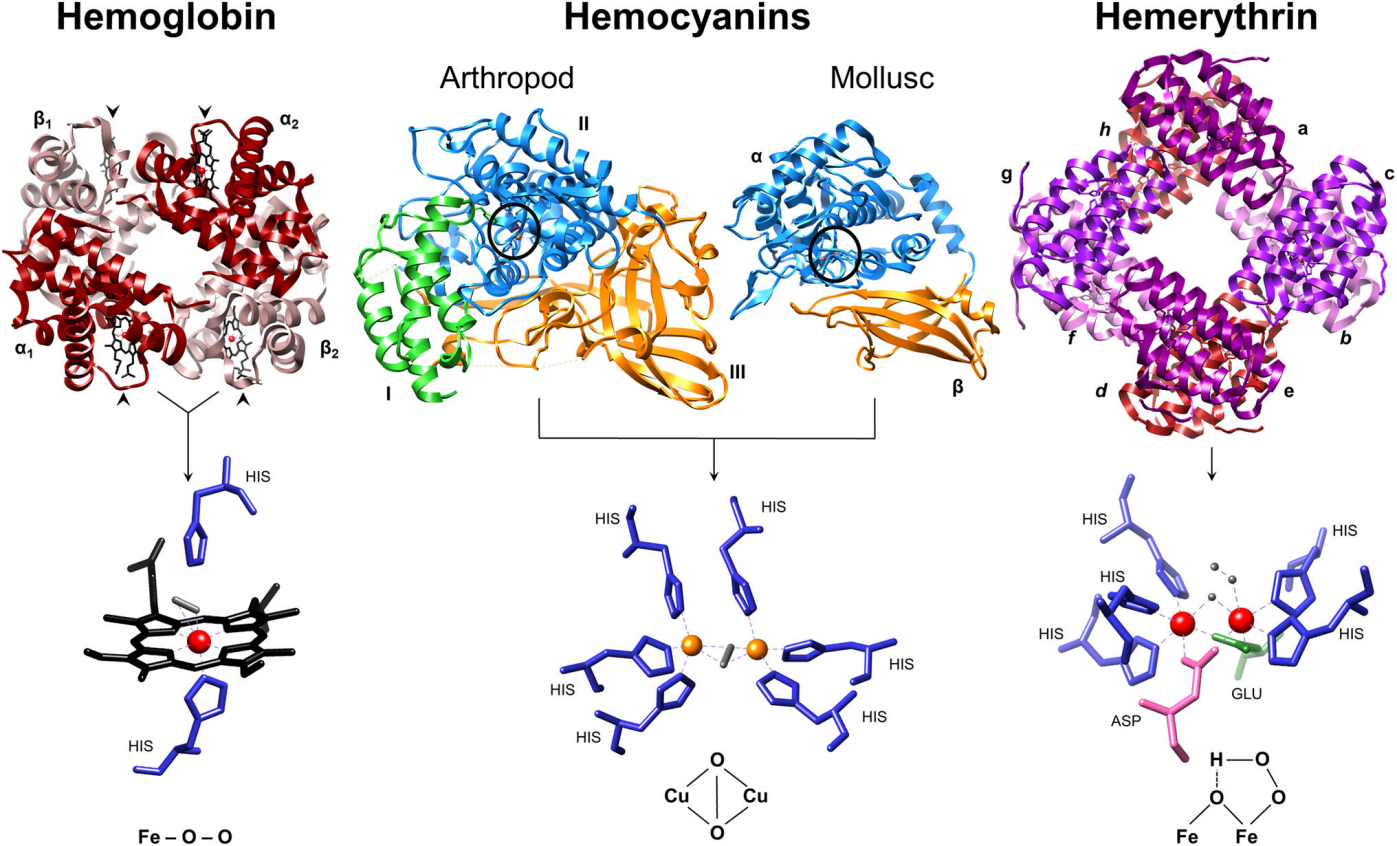
Three major classes of oxygen-transport proteins. Each of the 4 subunits (α1, α2, β1, β2) making up human Hb (and monomeric myoglobin) contain heme cofactors (Fe^2+^—protoporphyrin IX) that bind O_2_. Each heme group is indicated by an *arrow head*. The proximal histidine forms a direct bond with the iron atom, while the distal histidine is suggested to form a hydrogen bond with O_2_. The distal His hinders the energetically favoured straight binding of O–O. Arthropod hemocyanin subunits consist of three domains (*green*,* blue*,* orange*) and mollusc hemocyanin FUs consist of two domains (*blue*,* orange*). The* blue* domains possess two copper atoms, each is coordinated by three highly conserved histidine residues. O_2_ is bound in a ‘side on’ (μ–η^2^:η^2^) bridging formation between CuA and CuB. Hemerythrin (and myo-hemerythrin) secure O_2_ between two iron atoms (Fe_1_ and Fe_2_) which are held in place by five histidines residues, one aspartic acid and one glutamic acid. In the process, a hydroperoxide (OOH–) complex is formed. Images were produced using UCSF Chimera [202] and crystal structures from the Protein Data Bank: human hemoglobin tetramer 1GZX (α_2_β_2_, ~64 kDa), arthropod hemocyanin subunit 1OXY (~72 kDa), mollusc hemocyanin functional unit 1JS8 (~50 kDa) and sipunculid hemerythrin homo-octamer 1I4Y (~108 kDa). *Inset* oxygen is *coloured grey*; iron is *coloured red*; copper is *coloured orange*; histidines are *coloured blue*: aspartic acid is *pink* and glutamic acid is *green*

Vertebrate Hb consists of two identical α-chains and two identical β-chains with molecular masses of ~16 kDa each. Two αβ-dimers assemble in* C2* symmetry to form the Hb tetramer (~64 kDa) (Fig. 1). Individual subunits are comparable to monomeric myoglobin (Mb), and in all cases, Hb and Mb fold into a nest of α-helices [12]. Heme prosthetic groups are present in each subunit, consisting of a protoporphyrin ring and a single iron ion in the centre that is coordinated by the proximal histidine of α-helix F. At the other side of the heme, oxygen binds reversibly to the iron in an “end on” coordination (Fig. 1). Binding of oxygen to Hb induces a conformational rotation (15°) of one αβ-dimer against the other, thus switching from a tense (T) deoxygenated state to a relaxed (R) oxygenated state. Cooperative oxygen binding can be modulated by an allosteric effector such as 2,3-diphosphoglycerate in human Hb along the symmetry axis of the tetramer [12]. Vertebrate myoglobins (Mbs) are oxygen storage proteins in red muscle (e.g. cardiac) and other tissues, working to build up a pO_2_ gradient from blood vessels to mitochondria for ATP synthesis [13].

Extracellular Hbs (or erythrocruorins) are mostly large, oligomeric proteins with molecular masses up to 3.6 MDa. Vinogradov (1985) classified them into four separate groups: (a) single-domain, single-subunit Hbs (~16 kDa) found in trematodes and some insects, (b) two-domain, multi-subunit Hbs in branchiopod crustaceans such as *Daphnia* and *Triops*, (c) multi-domain, multi-subunit Hbs in carapace-free brachiopod crustaceans, the planorbid snails and some clams (~1.7 MDa) and (d) single-domain, multi-subunit Hb aggregates ca. 3.6 MDa in annelids [14]. The first resolved structure of a GE-Hb was from the earthworm, *Lumbricus terrestris* [6]. This mega-molecule consisted of 144 Hbs and 36 linker subunits assembled to form a core complex with* D6* symmetry. Recently, the quaternary structure of *Glossoscolex paulistus* plasma Hb was presented with a resolution of 3.2 Å, which is the highest resolution reported for a hexagonal bilayer Hb with 12 protomers [15].

### Hemocyanins

Although strikingly different in structural appearance, both arthropod and mollusc Hcs contain dicupric (histidine coordinated) groups that reversibly bind molecular oxygen in a side on (*μ* − *η* ^2^:*η* ^2^) bridging coordination [16] (Fig. 1). Arthropod Hc is composed of kidney-shaped subunits (~72 kDa, each with an oxygen binding site) arranged into hexamers (Fig. 1) [17]. Hexamers are formed when three subunits assemble back to back and dimerize isologously with a second trimer along the rotational axis (but are twisted against each other by 60°). Individual hexamers or multiples of hexamers have been observed in vitro, the largest of these is an 8 × 6 mer (~3.4 MDa) purified from horseshoe crab genera *Limulus* and *Tachypleus*. Invertebrate Hcs and GE-Hbs show strong hierarchies in structural organisation corresponding to hierarchical allosteric interactions (‘nesting’) [18, 19].

Hc concentration in the hemolymph varies greatly depending on the species, ~20–80 mg mL^−1^. In extreme cases, Hc content has been calculated in excess of 140 mg mL^−1^ in the chelicerate, *Limulus polyphemus* [16, 20]. Accumulating evidence suggests that Hc is an integral component of biological defence systems within arthropods [reviewed by 21]. Oxygen-carrying Hc can be activated by host (clotting proteins, phospholipids, AMPs, proteases, lipoproteins) and microbial (proteases, membrane ligands) factors to combat infection, parasitism, viremia and physical damages [22–32].

Mollusc Hcs are extremely large protein complexes dissolved in the hemolymph of gastropods and cephalopods [33, 34]. Typically, subunit molecular masses range from 330 to 450 kDa depending on the species. Subunits are composed of 7 or 8 (50 kDa) functional units (FU; Fig. 1), designated FU-a to FU-h. Ten of these subunits form a hollow cylinder with a diameter of ~310 Å and a height of ~160 Å. Decameric Hcs are found in cephalopods such as *Nautilus pompilius* [35]. Two of these cylinders can associate along the rotational axis to form di-decamers as observed in marine gastropods [33]. The largest known mollusc Hc is a 13.5 MDa tri-decamer discovered in several species of snail, e.g. *Melanoides tuberculata* and *Terebralia palustris* [36]. Renewed interests in Hc structural complexities and the assemblages of associated sugars and lipids aim to exploit the vast therapeutic potential of these megamolecules (Table 1). Especially from molluscs, Hcs are tested for application as bio-adjuvants (viral and bacterial antigens/haptens), immune-stimulants for treatment of cancers such as melanoma, and carrier molecules for vaccines (Table 1) [37–40].

**Table 1:** Recent examples of hemocyanin-based therapeutics

Source	Chemical modification	Adjuvancy, immune-stimulatory and anti-cancer properties	References
Blacklip abalone (*Haliotis rubra*)	Native	Antiviral: herpes simplex virus-1	[41]
Chilean abalone (*Concholepas concholepas*)	Native Oxidation of carbohydrates using sodium periodate	B16F10 melanoma model (IFN-γ secretion) Mouse bladder carcinoma model	[42] [37]
Giant Keyhole Limpet (*Megathura crenulata*)	Rindopepimut^a^ conjugated to KLH Heptavalent KLH and QS-21^b^ Sialyl-Tn KLH	Glioblastoma multiforme (brain) Epithelia ovarian, fallopian tube and peritoneal cancer Metastatic breast cancer	[43–45]
Limpet (*Fissurella latimarginata*)	Native Oxidation of carbohydrates using sodium periodate	Melanoma (B16F10 cells)	[38]
Shrimp (*Litopenaeus vannamei*)	Native	HeLa cells (cervical cancer)	[46]
Snails *(Helix aspersa)* *(Helix lucorum)* *(Helix pomatia)*	Native	Bladder cancer cell lines (T-24 and CAL-29) Ovarian carcinoma (FraWü) Acute monocytic leukaemia (THP-1) Human malignant glioma (LN-18) Human Burkitt’s lymphoma (Daudi) cell lines Colon carcinoma (murine model) Adjuvant for microbial and viral antigens	[47–49]
Whelk (*Rapana thomasiana*)	Choline amino acid salts Imidazolium-based amino acid ionic liquids	Human breast cancer cells (MCF-7)	[39, 40]

^a^Epidermal growth factor-related peptide

^b^Synthetic saponin adjuvant

### Hemerythrins

Hemerythrins (Hr) are relatively rare, non-heme, di-iron, dioxygen-binding proteins present in specialised coelomocytes (hemerythrocytes) of brachiopods, priapulids, sipunculids and annelids [50–53]. Most often viewed as an octamer of molecular mass ~108 kDa (Fig. 1), dimeric, trimeric and tetrameric isoforms of Hr have also been observed. These homo- or hetero-octamers are made up of α- and β-type subunits, each ~13–14 kDa in size [54]. Subunits consist of a four-α-helix motif that houses the two iron ions, one being hexa-coordinated and the other penta-coordinated (bridged by a hydroxyl ion). Between the hydroxyl group and a single iron ion, oxygen is bound reversibly in an ‘end on’ position (Fig. 1) [55]. To date, Hr has not been detected in a deuterostome, whereas many bacteria, fungi and archaea contain Hr-like domains that are seemingly involved in chemotaxis [56]. Muscle-specific hemerythrin (myoHr), which is functionally equivalent to Mb, has been observed in polychaete and sipunculid tissues [57, 58]. Uniquely, the phylum Annelida contains isoforms of all known iron-based OTPs [5, 51].

### Hemoglobin and erythrocytes contribute to mammalian innate immunity

**Fig. 2:**
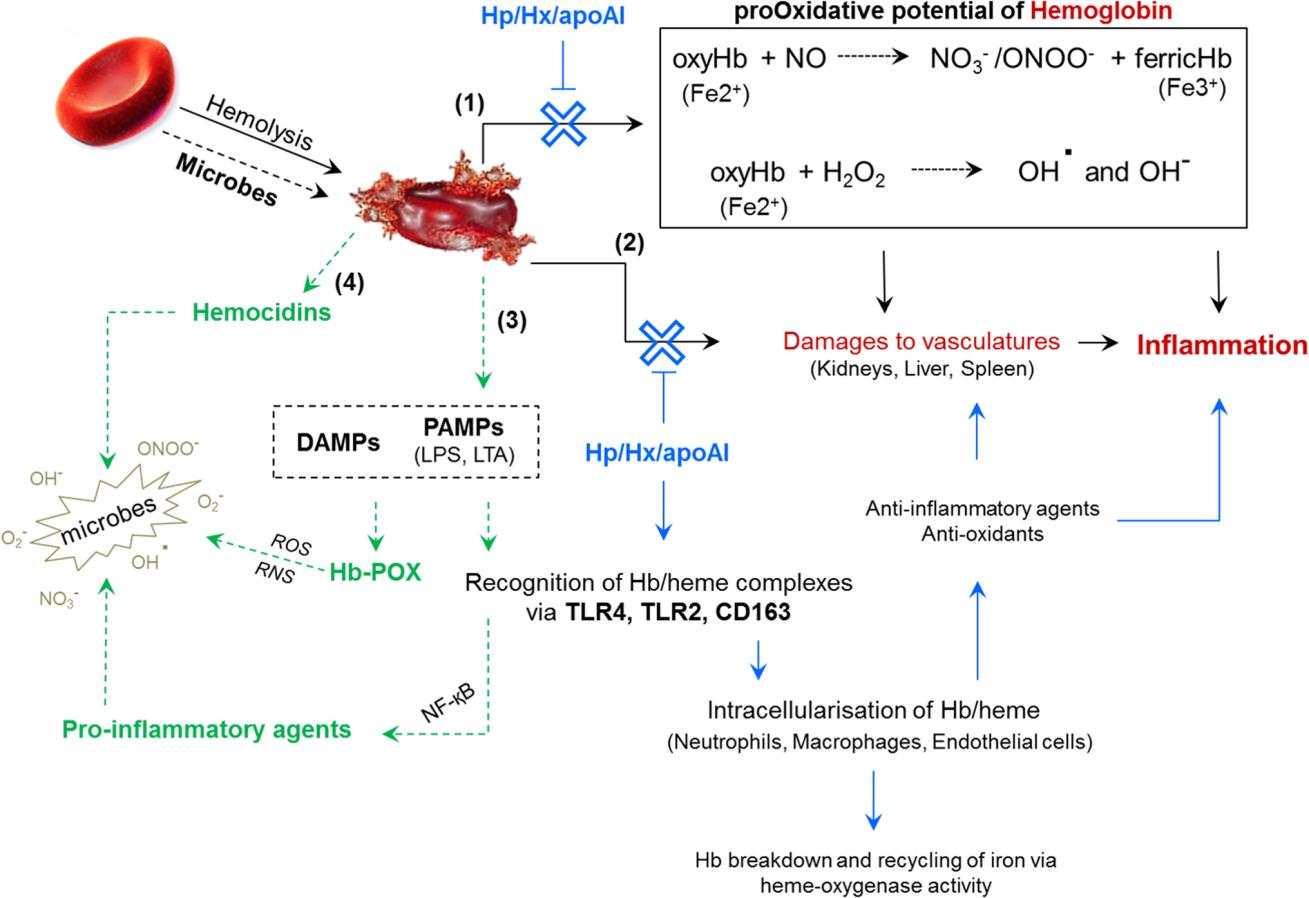
Schematic representation of hemoglobin functionality beyond oxygen transport. Hemolysis, whether it is caused by microbes or physical trauma, leads to the uncontrolled release of hemoglobins (Hbs). Extracellular Hb inflicts damages by producing reactive oxygen/nitrogen species (**1**) and interfering with hepatic, splenic and renal physiologies (**2**). Inflammation can be avoided/controlled by Hb**-**scavenging glycoproteins [haptoglobin (Hp), hemopexin (Hx) and apoplipoprotein A-I (apoAI)] and soluble receptors (CD163). These proteins intercept Hb, neutralise its oxidant properties, and direct it towards immune cells for degradation and to promote anti-inflammatory responses. Chemical (glutathione) and enzymatic antioxidants (superoxide dismutase, catalase) are recruited also. Anti-infective responses are triggered when Hb binds to PAMPs/DAMPs (**3**). Microbial ligand (PAMPs)–Hb complexes are recognised by immune cells whereupon pro-inflammatory molecules are released, and Hb is converted into a pseudoperoxidase (POX). If Hb has been enzymatically processed prior to erythrocyte rupture, then hemocidins (antimicrobial peptides) will also been disseminated (**4**). This scheme was produced by summarising information presented in the following manuscripts: [61, 62, 64, 68, 80]

Iron is a precious commodity utilised by microbes for growth and pathogenicity. To colonise and persist in metazoans, microbes must circumvent the many iron-withholding mechanisms of the innate immune response [reviewed by 60]. During infection, hemolytic bacteria (e.g. *Staphylococcus aureus* and *Streptococcus pyogenes*) will lyse erythrocytes to exploit the iron stored within. The extra-erythrocytic Hb is detected and sequestered by the glycoproteins, haptoglobin (Hp) and hemopexin, and the lipid-free apolipoprotein A-I, all of these are freely dissolved in the plasma [60–63]. Binding of Hp to Hb restricts access to the iron centre, thereby neutralising Hb’s pro-oxidative potential and avoiding damage to vasculatures [64]. Macrophages recognise the Hp/Hb complex via the CD163 receptor and internalise the proteins to prevent further inflammation (Fig. 2) [65]. The parasite *Trypanosoma brucei* uses a glycoprotein receptor to consume Hp/Hb complexes for iron removal and recycling. Humans take advantage of this trypanosome receptor by associating lytic molecules (high-density lipoproteins) with Hp-related proteins; tricking the parasite into ingesting the trypanolytic substance [66].

In severe cases of hemolysis (called hemoglobinemia), the excess concentration of Hb overwhelms the scavenging responses and can cause potentially fatal blockages in the kidneys [see review 62]. Extracellular Hb is a redox-sensitive molecule with the potential to generate reactive oxygen species (ROS) [67]. The ferrous (Fe^2+^) form can convert hydrogen peroxide (H_2_O_2_) into hydroxyl radicals (OH^.^) and anions (OH^−^) via Fenton’s reaction. Such ROS disrupt tissue and cellular integrity via the peroxidation of lipids and the oxidation of nucleic and amino acids [68]. Moreover, plasma oxy-Hb can bind to, and react with, nitric oxide (NO) to produce peroxynitrite (ONOO^−^) and ferric (Fe^3+^) oxidised Hb (methemoglobin) [64, 69, 70] (Fig. 2). NO is an essential antioxidant and plays key roles in immunity, neurotransmission and signalling. In mice and snails (*Biomphalaria glabrata*), resistance to *Schistosoma mansoni* is dependent on the production of NO by macrophages and hemocytes, respectively [71, 72]. NO possesses distinct anti-parasitic properties; therefore, the release of Hb could regulate biological defences targeting *Schistosoma* and *Plasmodium* species [73]. The digestion of blood by these hematophagous parasites yields an insoluble, crystalline Hb-derived product called hemozoin [74]. If hemozoin is not removed from circulation by the spleen and liver, it can be phagocytosed by circulating leukocytes. Accumulation of hemozoin in monocytes is said to interfere with key immune molecules such as protein kinase C and major histocompatibility complex II [75].

Traditionally, pro-inflammatory responses are mediated through the binding of pathogen-associated molecular patterns (PAMPs) by soluble and cell-associated pathogen recognition receptors (PRRs) [76]. PAMPs tend to be extracellular microbial cell wall components (LPS, LTA and β-glucans) and their degenerated membrane fragments. Cell-free Hb is categorised as a damage/danger-associated molecular pattern (DAMP) because it is perceived as an intracellular-derived indicator of pathological traumas such as malaria, sepsis and sickle-cell anaemia [77]. Not only is the Hb oligomer/dimer considered a DAMP, but the heme group (protoporphyrin ring) is recognised independently as an ‘alarmin’ [reviewed by 78]. Proteolytic digestion of extracellular Hb enhances the dissemination of labile heme within the blood, a physiological indicator of cystic fibrosis [68]. The control and removal of naked heme (hemin) from circulation is addressed by the protein hemopexin [62]. Several studies have noted Hb’s ability to interact with microbial ligands (PAMPs), Toll-like receptors (TLRs) and other DAMP molecules (e.g. heat-shock protein HMGB1) [24, 61, 68, 79–81]. Surface plasmon resonance revealed LPS binding sites are present on both the α and β globin chains of Hb [82]. Synthetic peptides representative of these putative ligand-binding regions targeted the lipid A moiety of LPS in vitro, and in doing so, disarmed the endotoxicity. Binding of LPS and/or LTA induces a conformational switch in Hb that causes the structure to loosen somewhat and enable peroxidase activity [24, 82]. Methemoglobin (metHb) alone and in combination with LTA can be recognised by TLR-2 on the neutrophil plasma membrane [81]. Such interactions enhance neutrophil function, initiating an NF-κB signal transduction cascade that culminates in the synthesis of cytokines and other pro-inflammatory agents. Endothelial cells can also detect extracellular Hb via a TLR-4 pathway [83] (Fig. 2).

Many studies (mentioned above) categorised extracellular Hb as harmful to the host and should be removed from circulation before noxious radicals are dispersed. Having said that, plasma Hb is an important warning to white blood cells (WBCs) that homeostasis has been compromised, and recently, Bahl et al. outlined a novel role for Hb in blood coagulation [84]. Macrophages responded to the presence of cell-free Hb by triggering the expression of the vertebrate pro-clotting initiator, tissue factor. Binding of Hb to tissue factor provided it with protection against antioxidants (e.g. glutathione), and reciprocally, the pro-oxidative potential of Hb was suppressed to mitigate collateral damage to the host’s cells. Infection-induced hemolysis and the liberation of Hb promote downstream pro-inflammatory and pro-clotting reactions [84] (Fig. 2). Remarkably, Hb gene expression and protein synthesis were recorded in cytokine (IFNγ) and LPS-stimulated murine macrophages [85] and surfactant-producing human alveolar type II epithelial cells [86]. The biological function of Hb production outside of erythroid tissues remains unclear. It is postulated, however, Hb may enable these particular cells to cope with nitrosative/oxidative imbalances as macrophages produce excess NO when presented with microbes, and alveolar epithelia are subjected to high levels of CO_2_ during gas exchange, which may affect cytosolic pH.

**Fig. 3:**
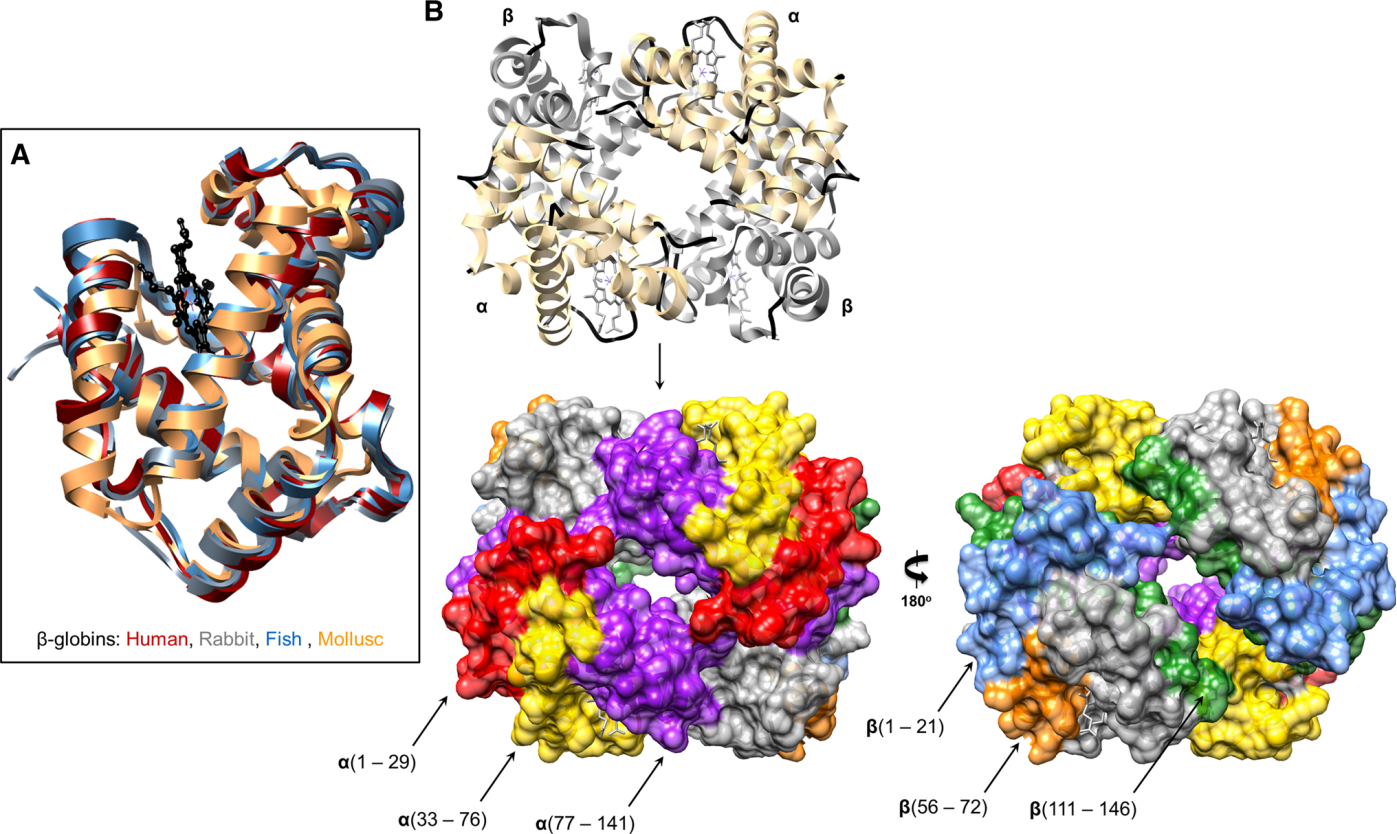
Hemoglobins and the locations of hemocidins. **a** Human (PDB 1GZX *red*), rabbit (PDB 2RAO *grey*), fish (PDB 3BJ2 *blue*) and mollusc (PDB 4HRR *brown*) β-globin structures have been aligned and superimposed to highlight the conserved helical structures. The protoporphyrin ring is coloured *black*. **B** Secondary structural features of the oxy-hemoglobin tetramer (PDB 1GZX) are presented as ribbons. Alpha and beta chains are *coloured light yellow* and *grey*, respectively. Coils are *coloured black*. The space-filling models of Hb were used to highlight the location of various antimicrobial peptides (*black arrows*). The α-chain peptides consist of residues 1–29 (*red*), 12–29 (*red*), 33–76 (*yellow*) and 77–141 (*purple*). The β-chain peptides consist of residues 1–21 (*blue*), 9–21(*blue*), 56–72 (*orange*) and 111–146 (*green*). It is worth noting that the Hb-β peptide 111–146 was detected in the placenta and in the cytosol of erythrocytes [94]

Beyond immune cell communication and hemostasis, Hb participates in host defences by releasing AMPs [87], discharging ROS locally [24], and functioning as a microbiostatic molecule (Table 2). The earliest record of Hb’s immune competence was reported over 55 years ago [9]. Hb prepared from human tissue extracts was inhibitory to several enteric bacteria (listed in Table 2), with maximum activity occurring at 37 °C under acidic conditions (pH <5.5) and low salt concentrations (<0.2 M). Bovine, equine, murine and rabbit Hbs were similarly antiseptic. Hb tetramers (~64 kDa) are probably too large to penetrate the bacterial membrane directly. The basic charge of Hb would promote non-specific electrostatic interactions with the acidic moieties of microbial polysaccharides, proceeding to immobilise the cells and prohibit replication. Two consecutive studies by Mak et al. [88] and Parish et al. [89] provided detailed accounts on the conversion of Hbs, Mb and cytochrome c into antimicrobials. Intact Hb and Mb were moderately effective at killing bacteria such as *E. coli* until the removal of the heme cofactors and partial unfolding of the proteins resulted in a broader spectrum of microbicidal properties and LD_50_ (µM) values comparable to conventional AMPs. By treating apomyoglobin and apohemoglobin with cyanogen bromide, the globin chains were deconstructed into AMPs ca. 50 amino acids in length (Table 2; Supp. Table 1; Fig. 3). This family of Hb-derived AMPs was referred to as ‘hemocidins’ [88]. Prior to the discovery of hemocidins, human Hb was already known to be a rich source of over 150 regulatory peptides, e.g. hemorphins with opioid-like tendencies [90]. In fact, the first Hb-derived AMP was removed from the gut of the cattle tick* Rhipicephalus* (*Boophilus*)* microplus* [91]. This 3.2 kDa AMP was identical to residues 33–61 (FLSFPTTKTYFPH-FDLSHGSAQVKGHGAK) of bovine α-Hb (Supp. Figure 1), and targeted Gram-positive bacteria, filamentous fungi and yeast [91]. A second tick species, *Ornithodoros moubata,* contained two anti-Staphylococcal peptides in its midgut after a blood meal [92]. Edman degradation verified the origin of these peptides to be overlapping fragments (residues 1–11; 3–19) from rabbit αHb. When in solution, the bovine peptides Hb33-61 and Hb1-23 do not form distinct secondary structures, i.e. they are unfolded. Upon insertion of Hb33-61 into anionic detergent micelles, the cationic peptide establishes an N-terminal β-turn and a C-terminal α-helical arrangement [93]. A flexible region, Pro44-Leu48, forms between the two structural motifs and may act like a hinge to help the peptide penetrate/rupture the lipid bilayers of microbes.

**Table 2:** Anti-infective activity of hemoglobins and myoglobin (including cryptides)

Organism	Conformational state	Size (kDa)	Charge	Activity range (MIC or LD_50_)*	References
**Hemoglobin**					
Alligator (*Alligator mississippiensis*)	Hemoglobin tetramer (α_2_β_2_), α-chain, β-chain	~68		**Antibacterial:** Gram− *Escherichia coli* (25–100 µg ml^−1^) *Pseudomonas aeruginosa* (25–350 µg ml^−1^) **Antifungal:** *Candida albicans* (20–150 µg ml^−1^)	[89, 133]
Blood cockles (clams) (*Scapharca kagoshimensis*) (*Tegillarca granosa*)	Hemoglobin dimer and tetramer Microbial challenge leads to substantial expression of Hb mRNA, peaking at 12 h. PSVQDAAAQISADVKK VLASLNFGDR ISAAEFGK ISAEAFGAINEPMK GHAITLTYALNNFVDSLDDPSR MGSYYSDECAAAWAALVAVVQAAL LNGHGLTLWYGIQNFVDQLDNADDLEDVARK	<60 1.6 1.1 0.8 1.5 2.4 2.5 3.5	Neutral Neutral Neutral Anionic Anionic Anionic Anionic	**Antibacterial:** Gram +/− *E. coli* *Vibrio parahaemolyticus* (11–100 µg ml^−1^) *Vibrio alginolyticus* (12–200 µg ml^−1^) *Vibrio harveyi* (1–200 µg ml^−1^) *Bacillus subtilis* (<180 µg ml^−1^) *Bacillus firmus* *Staphylococcus aureus* (>370 µg ml^−1^) *Micrococcus tetragenus* (<47 µg ml^−1^) **Ligand binding:** Lipopolysaccharide and peptidoglycan	[139–141, 144, 145]
Catfish (*Ictalurus punctatus)*	**HbβP-1**: AAKFGPSVFTPEVHETWQKFLNVVVAALGKQYH The Hb-AMP was expressed in skin and gill epithelium when challenged with *I. multifiliis*	3.7	Cationic p*I* = 9.22	**Anti-parasitic:** *Amyloodinium ocellatum* (54 µM) *Ichthyophthirius multifiliis* trophont (1.7 µM) *Tetrahymena pyriformis* (6.8 µM) **Antibacterial:** Gram− *Escherichia coli* (3.4 µM) *Vibrio alginolyticus* (13.5 µM) *Aeromonas hydrophila* (3.4 µM)	[119, 120]
Cow^a^ (*Bos taurus*)	Hemoglobin tetramer (α_2_β_2_) FLSFPTTKTYFPHFDLSHGSAQVKGHGAK (αHb: 33-61) Found in stomach of cattle tick (*Rhipicephalus microplus*)^b^ FLSFPTTKTYFPHFDLSHGSAQVKGHGAK **-NH**_**2**_ VLSAADKGNVKAAWGKVGGHAAE (αHb: 1-23) (α–Helix and unordered structures) VNFKLLSHSLLVTLASHL (αHb) Hb40-61a: KTYFPHFDLSHGSAQVKGHGAK	~68 3.2 2.2 ~2 2.4	Cationic p*I* = 10.12 Cationic p*I* = 9.44 Cationic p*I* = 9.69 Cationic p*I* = 10.12	**Antibacterial:** Gram +/− *Micrococcus luteus* (5–671 µM) *Staphylococcus epidermidis* (21 µM) *Escherichia coli* (IC_50_ = 0.1 µg ml^−1^) **Antifungal:** *Candida albicans* (5 µM) *Aspergillus nidulans* (1.3 µM) *Saccharomyces cerevisiae* (11 µM)	[9, 91, 93, 100–105, 148–150]
Crocodile^a^ (*Crocodylus siamensis*)	Hemoglobin tetramer (α_2_β_2_), α-chain VLSSDDKCNVKAVWCKVAG KVAGHLEEYGA WHKVDVAH HEAVNH ASFGEAVKHLDSIR VVVAIHHPGSLTPEVHASLDKF AIHHPGSLTPEVHASLDKFL AAHYPKDFGL	~68 2 1.2 ~1 0.7 1 2.35 2.2 1.1	Cationic Neutral Cationic Cationic Neutral Cationic Cationic Cationic	**Antibacterial:** Gram + *Bacillus amyloliquefaciens* *Bacillus subtilis* *Bacillus pumilus* *Bacillus megaterium*	[130, 131]
Guinea pig (*Cavia porcellus*)	Hemoglobin tetramer (α_2_β_2_)	~68		**Antibacterial:** Gram − *Escherichia coli* (IC_50_ = 0.02 µg ml^−1^)	[9]
Human^a^	Hemoglobin tetramer (α_2_β_2_), monomers (α & β), and apoglobins HbA1-29 (12–29): VLSPADKTNVKAAWGKVGAHAGEYGAEAL HbB1-21 (9–21); VHLTPEEKSAVTALWGKVNVD hHEM-β (111–145): VCVLAHHFGKEFTPPVQAAYQKVVAGVANALAHKYH (Present in erythrocytes and in placental blood) Foetal Hb-γ: WQKMVTAVASALSSRYH		Cationic p*I* = 7.54 Neutral p*I* = 6.23 Cationic p*I* = 9.26 Cationic p*I* = 10.45	**Antibacterial:** Gram +/− *Bacillus subtilis, Lactobacillus acidophilus, Salmonella spp.* (LD_50_ = ~5 µM)*, Staphylococcus aureus* (LD_50_ = ~7 µM)*, Staphylococcus carnosus, Escherichia coli* (1–20 µg ml^−1^), *Shigella sonnei, Micrococcus luteus, Enterococcus faecalis* (LD_50_ = 6.5–8 µM)*, Pseudomonas aeruginosa* (LD_50_ = 4.4–6.7 µM) and *Klebsiella spp.* (LD_50_ = 5.8–8.3 µM) **Antifungal:** *Saccharomyces cerevisiae, Candida albicans* (LD_50_ = 6.25–14 µM)*, Candida parapsilosis* (LD_50_ = 6.25–12 µM)*, Candida krusei* (LD_50_ = 12.5–25 µM) **Ligand binding:** lipopolysaccharides	[9, 80, 88, 94–99]
Horse (*Equus ferus caballus*)	Hemoglobin tetramer (α_2_β_2_)	~68		**Antibacterial: **Gram− *Escherichia coli* (IC_50_ = 0.4–2 µg ml^−1^) **Antifungal: ** *Candida albicans* (MIC = 250 µg ml^−1^)	[9, 89]
Indian major carp (*Cirrhinus mrigala*)	Hemoglobin monomers (α & β),	~16		**Antibacterial:** Gram− *Pseudomonas aeruginosa, Salmonella typhi, Salmonella paratyphi and Vibrio cholera*	[127]
Japanese eel (*Anguilla japonica*)	FAHWPDLGPGSPSVKKHGKVIM	2.4	Cationic p*I* = 10.3	**Antibacterial: **Gram +/− (10.5–84 µM) *Edwardsiella tarda, Aeromonas sp., Aeromonas hydrophila, Vibrio alginolyticus,, Vibrio parahaemolyticus, Micrococcus luteus and Staphylococcus aureus*	[124]
Mouse	Hemoglobin tetramer (α_2_β_2_)	~68		**Antibacterial:** Gram− *Escherichia coli* (IC_50_ = 0.1 µg ml^−1^)	[9]
Rabbit (*Oryctolagus cunniculus*)	Hemoglobin tetramer (α_2_β_2_) VLSPADKTNIK SPADKTNIKTAWEKIGS Peptides were found the in stomach of soft tick (*Ornithodoros moubata* ^)^	~68 1.2 1.9	Cationic p*I* = 9.53 Cationic p*I* = 9.45	**Antibacterial: **Gram +/− *Escherichia coli* (IC_50_ = 0.02 µg ml^−1^) *Staphylococcus aureus*	[9, 92]
Rat	Hemoglobin tetramer (α_2_β_2_)	~68		**Antibacterial:** Gram− *Escherichia coli* (IC_50_ = 0.02 µg ml^−1^)	[9]
Sea bass (*Dicentrarchus labrax*)	Hemoglobin-like protein (mRNA expression)			**Antibacterial:** Gram− *Vibrio anguillarum*	[122]
Snake (*Thamnophis sirtalis*)	Hemoglobin tetramer (α_2_β_2_)	~68		**Antibacterial:** Gram +/− *Streptococcus faecalis* (15 µg ml^−1^) *Escherichia coli* (10 µg ml^−1^) **Antifungal:** *Candida albicans* (≥100 µg ml^−1^)	[89, 133]
Stingray (*Potamotrygon henlei*)	β-Globin chain	~16		**Antibacterial:** Gram+/− *Escherichia coli* (MIC; 12 µM) *Micrococcus luteus* (MIC; 4 µM) **Antifungal:** *Candida tropicalis* (MIC; 4 µM)	[126]
Tuna (*Katsuwonus pelamis*)	TQQAFQKFLAAVTSALGKQYH	2.3	Cationic p*I* = 9.7	**Antibacterial:** Gram+/− *Bacillus subtilis, Staphylococcus aureus, Streptococcus iniae* (MEC: 6.5–57 µg ml^−1^), *Salmonella enterica, Pseudomonas aeruginosa, Salmonella enterica, Shigella sonnei, Vibrio parahaemolyticus, Escherichia coli* (MEC: 2–19 µg ml^−1^) **Antifungal: ** *Candida albicans* (MEC: 12 µg ml^−1^)	[125]
**Myoglobin** ^a^					
Horse Whale *(Physeter catodon)*	Mb **56–131** Apomyoglobin Myoglobin fragments: 1–55, **56–131** and 132–153^c^	~17		**Antibacterial:** Gram+/− *Escherichia coli* (LD_50_ = 3.1 µM), *Salmonella* serotype *Krefeld* (LD_50_ = 4.7 µM), *Pseudomonas aeruginosa* (LD_50_ = 7.9 µM), *Klebsiella oxytoca* (LD_50_ = 4.2 µM), *Enterococcus faecalis* (LD_50_ = 7.9 µM), *Staphylococcus aureus* (LD_50_ = 2.6 µM) **Antifungal:** *Candida albicans* (LD_50_ = 11 µM)	[88, 151]

* Minimum inhibitory concentration (MIC) or LD_50_ values were not available for all (poly)peptides listed

^a^An extended list of hemoglobin/myoglobin-derived peptides is available as supplementary material, Table S1

^b^Formerly *Boophilus microplus*

^c^Cyanogen bromide treatment

To date, natural sources of human hemocidins include, but may not be limited to, placental tissue, erythrocytes and menstrual vaginal secretions [87]. Liepke et al. observed two AMPs from placental tissue, one from γ-Hb (130–146) and a second from β-Hb (111–146). The latter βHb peptide was present in large amounts, ~360 mg kg^−1^ tissue, pre-processed from the Hb tetramer within the cytosol of erythrocytes, and could bind to endotoxins [94]. The most convincing evidence for hemocidins functioning in vivo comes from vaginal blood [95–99]. Initial screenings identified 44 hemocidins, most of them originating from the N-terminus of αHb. Two synthetic peptides, Hbα 35–56 and Hbβ115–146, identical to natural peptides purified previously showed preferential activity towards Gram-negative bacteria, less activity toward Gram-positives, and no effect on fungal growth/reproduction [95]. Hbβ115–146 is an acidophilic, halo-tolerant peptide that potentiates the microbicidal effects of common neutrophil-derived immune effectors such as α/β-defensins and lysozyme, which are found in the female urogenital tract [97]. Hemocidins present in the vagina act as bacterial deterrents and assist immune defences during times of intense physiological strain, such as menstruation and childbirth [87, 96]. It remains unclear how hemocidins are formed and deposited into the vagina. The acidic pH may denature the Hb oligomers enough to allow endo/exo-peptidases and neutrophil-derived matrix metalloproteases to separate the peptides in a step-wise manner.

The laboratory of J.L. Ding in Singapore has provided unequivocal evidence to support a role for Hb in innate immunity. Damage to, and lysis of erythrocytes, by hemolytic bacteria guides the release of pro-oxidative Hb into the surrounding milieu. The more virulent pathogens continue to secrete proteases for digesting Hb. Microbial ligands and proteases act in synergy and amplify the pseudoperoxidase (POX) activity of Hb. Concurrently, the pathogen is bombarded with a battery of ‘dual-active’ Hb congeners/peptides that bind to exoplasmic membrane structures and inflict localised cytotoxic radicals to weaken/kill the pathogen in situ [24, 80, 82]. Hb-PAMP aggregates are inflammatory agonists (mentioned above), stimulating the expression of cytokines before being recycled by proteasomes and heme-oxygenase within circulating phagocytes (Fig. 2).

Functionally versatile hemocidins have been retrieved from endogenous sources (erythrocytes, ticks, uterine secretions) and by in vitro chemical/enzymatic manipulation (e.g. amidation) of commercially sourced globins (listed in Table 2; Supp. Table 1). Synthetic analogues of bovine and human Hb peptides are now screened routinely for therapeutic potential, e.g. anti-HIV-1 [94, 100–106]. Peptide release from larger ‘maternal’ proteins is more widespread than once thought. Indeed, the ‘cryptome’ refers to the entire subset of proteins/peptides released from maternal sources, which have alternative or heightened activities [107–109]. Human lactoferrin [110, 111], lysozyme [112] and cathepsin G [113] are more examples of macromolecules containing encrypted peptides.

## Use of hemoglobin by microbes

Certain pathogens adapted for intracellular life enlist their own heme containing globins to combat the harsh internal environment of leukocytes. A truncated Hb produced by *Mycobacterium tuberculosis* (HbN) is necessary for infectivity [114]. The microbe-derived HbN decomposes NO produced by macrophages and neutrophils so the bacterium may survive within the cytosol. HbN acts as a NO dioxygenase despite the absence of a true reductase domain [115]. HbN expression intensifies once the bacterium enters the WBC. Subsequently, the protein is glycosylated post-translationally and localised to the cell membrane/wall. The expression of CD80/86 co-stimulatory surface receptors on the phagocyte cell surface is suppressed during *M. tuberculosis* occupancy, linked to an increase in HbN concentration [114]. It appears that HbN not only protects the bacterium from the cytosolic defences of WBCs, but also modulates the expression of host immune factors.

The causative agent of thrush, *Candida albicans,* secretes up to ten acid hydrolases (aspartic peptidases) when attempting to colonise the vagina. These enzymes attack the host’s defences, and are capable of using the existing Hb as a substrate for peptide production. These Hb hydrolysates are effective bactericidals, especially in the presence of *Lactobacillus acidophilus* [99]. Intriguingly, these observations imply that *C. albicans* exploits human Hb to antagonise bacteria and reduce competition within the vagina.

### Fish hemoglobin and erythrocytes

Functional plasticity of erythrocytes from non-mammalian vertebrates such as trout (*Oncorhynchus mykiss*) and chickens has been confirmed in vitro [116]. Aside from links to reproductive and endocrine physiologies, these nucleated erythrocytes employ common PRRs (e.g. TLR 3) to detect PAMPs (LPS, PGN) and respond by synthesising a plethora of immune-related mRNAs (chemokine CCL4, IFNα). These erythrocytes are further capable of communicating the presence of viral mimics (polyinosinic: polycytidylic acid) to macrophages via a type-1 IFN response [116]. Erythrocyte–pathogen antibiosis is frequently encountered across the literature, yet critically, there is now sufficient evidence for erythrocyte-specific roles in fish, reptile and bird innate immunity [reviewed by 117]. First, in 2004, antibacterial proteins were recovered from ruptured erythrocytes of rainbow trout [118]. These cationic proteins targeted *Planococcus citreus* and *E. coli* with MIC values in the sub-micromolar range. Although the authors did not purify individual compounds, they speculated (based on size) that histone H2A was involved. It is highly likely some Hb found its way into the final protein extract, regardless of the ‘harsh’ extraction procedure used and the loss of Hb via precipitation [118]. Lately, piscine Hb subunit chains (α & β) and peptides of various sizes have been reported acting in an anti-infective manner (listed in Table 2).

**Fig. 4:**
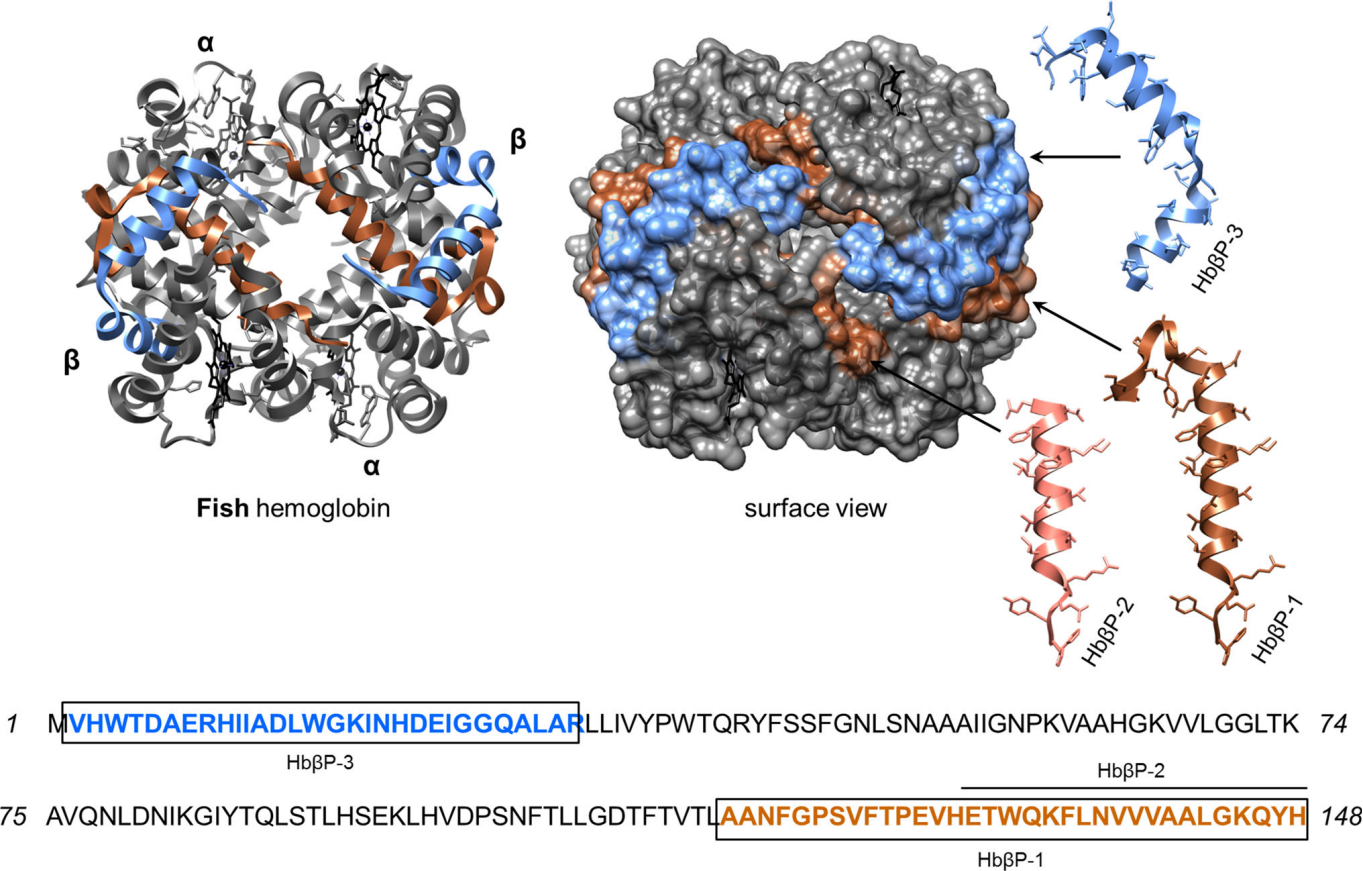
Hemoglobin-derived antimicrobial peptides from fish. The overlapping encrypted peptides (HbβP-1, 2 and 3) of fish (*Ictalurus punctatus;* GI:318171215) hemoglobin are presented using the crystal structure of rainbow trout hemoglobin (3BOM). The helical structures of each peptide are presented as *ribbons*, and their locations are indicated by *black arrows*. It has not been confirmed whether the peptides retain these structural features upon detachment from the Hb

In catfish (*Ictalurus punctatus*) infected with the ciliate parasite ich (*Ichthyophthirius multifiliis*), variants of the β-Hb gene were transcribed and translated in epithelial surfaces of the skin and gills, as well as in erythrocytes [119]. In total, three cationic Hb-derived peptides were identified, HbβP-1 to HbβP-3. These peptides emerged from both the C- and N-termini of the β-globin monomer (Fig. 4). The extra-erythrocytic peptide, HbβP-1 (3.7 kDa), was lethal to eukaryotic and prokaryotic fish ectoparasites: *Amyloodinium ocellatum*, *I. multifiliis* and *Tetrahymena pyriformis* [119, 120]. In vivo concentrations of HbβP-1 increased under immune challenge and were selectively toxic to the trophont stage of the parasites. Unlike many of the human hemocidins discussed previously, antimicrobial properties of HbβP-1 were limited to a few Gram-negative bacterial pathogens (Table 2).

Significant increases in Hb mRNAs were detected in several tissues (gill, skin and spleen) of European sea bass, *Dicentrarchus labrax,* resulting from exposure to acute physical stress (crowding) and pathogenic challenge (*Vibrio anguillarum*) [121, 122]. This study mirrored patterns of Hc up-regulation in the hepatopancreas of shrimp (*L. vannamei*) subjected to microbial and thermal stresses [21, 123]. The first fish α-Hb peptide was extracted from the liver of Japanese eel (*Anguilla japonica* [124]), and following that, a β-Hb peptide was removed from the liver of Tuna (*Katsuwonus pelamis* [125]). Peptides from each fish were <2.5 kDa in size, positively charged and composed of α-helices. Antimicrobial activity of the tuna Hb peptide (SHβAP) was heat stable and pH resistant, but was non-functional in the presence of chymotrypsin and trypsin [125]. C-terminal amidation of SHβAP enhanced its activity, perhaps by altering the electrostatic interactions with the anionic bacterial membranes. Generally, post-translational modification of peptides increases their metabolic stability against endogenous peptidases.

The biomolecular composition of stingray (*Potamotrygon henlei*) mucus was interrogated for the presence of antimicrobials, wherein a β-Hb polypeptide (~16 kDa) was classified [126]. In vitro assays revealed non-specific microbicidal actions of this β-Hb monomer in the presence of bacterial and fungal targets (Table 2). The stingray Hb did not show any adverse cytotoxicity upon exposure to mammalian cells, and injection of the protein (10 µM) into the cremaster venule of mice induced an ephemeral response in leukocyte rolling behaviour (visualised using intravital microscopy). These data suggest β-Hb is a putative immune bioactive from stingray mucus and has potential therapeutic applications in humans. The Indian major carp, *Cirrhinus mrigala*, also contains biologically active α- and β-Hb chains in skin mucus, as well as histones H2A, H3 and H4 [127]. The immune activity of Hbs in fish mucus and skin epithelia serves as a first-line defence against parasites and pathogens.

### Reptile hemoglobin

Despite crocodiles spending most of their lives in dirty, microbiologically hazardous waters, they show few signs of severe infection even when seriously wounded [128, 129]. This resistance is due, in part, to the multi-functionality of Hb. Irrespective of the presence or absence of the heme prosthetic group, crocodile (*Crocodylus siamensis*) Hb tetramers, various degenerated fragments (<21 amino acids), individual globin units (α and β), and synthetic α-Hb monomers were all capable of killing Gram-positive bacteria (four species of *Bacilli*) but appeared ineffective against Gram-negative bacteria (*E. coli*) [130, 131]. Electron micrographs taken of *B. subtilis* [ATCC 6633] cultured in the presence of purified Hb fractions depicted cell membrane irregularities within 2 h. Many of these crocodile hemocidins share typical features of AMPs: net-positive charge, >30 % hydrophobic content and their predicted secondary structural motifs are dominated by α-helices (Table 2; Table S1). The latter is not entirely surprising considering the conserved helical arrangement of all known Hbs (Fig. 3). Crocodile Hb is a potent scavenger of oxygenic radicals in vitro, albeit the significance of this antioxidant role in vivo has yet to be explored [132].

The antimicrobial features of ectotherm Hbs are not restricted to Gram-positive bacteria, as snake (*Thamnophis sirtalis*) and alligator (*Alligator mississippiensis*) Hbs can inhibit the growth of *E. coli*, *Pseudomonas aeruginosa* and the pathogenic yeast, *C. albicans* [89, 133] (Table 2). Alligator Hb failed to suppress Gram-positive bacteria, *Streptococcus faecalis* and *S. aureus,* using a disc diffusion approach [89]. The potency of alligator Hb differs between each globin subunit, e.g. up to fivefold less αHb (MIC = 30 µg mL^−1^) was needed to inhibit yeast compared to βHb (MIC = 150 µg mL^−1^) [133].

## An emerging role for invertebrate (bivalve) hemoglobin in innate immunity

Almost all cephalopods and gastropods utilise Hc to dispense dioxygen to metabolically active tissues; an exception being freshwater snails. Species such as the planorbid snail, *Biomphalaria glabrata,* use giant extracellular Hbs (1.44 MDa) to meet their respiratory needs in what is considered to be an evolutionary abandonment of Hc [134, 135]. In blue-blooded (Cu) and red-blooded (Fe) snails, Hc and Hb are synthesised inside specialist rhogocytes (pore cells) and then released into the plasma [136, 137]. Bivalves lack Hc, instead they utilise cell-bound Hbs which are structurally similar to vertebrate Hbs [5, 138]. Analogous to fish and reptiles, bivalves store their Hb within nucleated erythroid-like cells. Trematode infestation of the Sydney cockle, *Anadara trapezia*, induced measureable increases in circulating erythrocyte numbers, over double compared to non-parasitised animals [138]. These data add support to earlier findings demonstrating the immune competence of erythrocytes [117].

The cDNA (748 bp) of an intracellular homo-dimeric HbI (~31 kDa) from the blood clam *Tegillarca granosa* was cloned and sequenced to reveal ~82 % similarity with Hbs from *Scapharca kagoshimensis* and *Scapharca inaequivalvis* [139]. Messenger RNAs of HbI were expressed constitutively in the hemocytes, adductor muscle, foot, gills, gastrointestinal tract and mantle. When clams were injected with LPS, *Vibrio parahaemolyticus* and/or PGN, HbI mRNA transcript numbers increased significantly. The highest levels of expression were detected in the hemocytes, with an 800-fold increase at 12 h post-infection (h.p.i.) compared to the control groups. Differential temporal expression patterns of *T. granosa* HbII-A and -B genes in hemocytes were also detected in the presence of microbial ligands (LPS and PGN) and intact bacteria [140, 141]. Over 20 individual nucleotide polymorphisms have been identified across all three *T. granosa* Hb genes [141]. Polymorphic loci at exon2–146 (serine to proline switch) and exon2–23 (alanine to threonine switch) on HbII-A and HbII-B, respectively, were recovered from clams having survived heavy *V. parahaemolyticus* loads. These amino acid substitutions likely confer alternate functionality to newly synthesised Hbs. Similarly, 13 Hc-associated SNPs have been identified in shrimp (*L. vannamei*) infected with the same pathogens [142, 143]. These molecular alterations were located in the immunoglobulin-like domain and C-terminal region of the shrimp Hc resulting in improved microbial agglutination properties.

Positive microbicidal activities of intact *T. granosa* HbI and HbII were observed in the presence of *E. coli* and several Gram-positive bacteria. Seven Hb-derived peptides ranging in size from 0.8 to 3.5 kDa (Table 2) were effective against Gram-negative bacteria only, verified using live/dead cellular staining [144]. These bivalve hemocidins were removed from the Hb protomers via trypsin digestion and purified to homogeneity. The aquatic pathogen, *Vibrio harveyi,* was particularly sensitive to the neutral Hb peptide 1 (PSVQDAAAAQISADVKK), with an MIC value of 1 µg mL^−1^. Each Hb-derived peptide demonstrated antibacterial potential (Table 2). Additionally, Hb oligomers from *S. kagoshimensis* proved efficient at killing Gram-positive bacteria, yet had no measureable effect on fungal moulds (*Aspergillus niger*, *Penicillium glaucum*) or any Gram-negative bacteria tested [145]. The authors of these studies considered ROS production by peroxidase and PO-like activities of Hb were likely contributing factors to the antimicrobial mechanism. The use of POs to produce ROS and melanins is a conserved defence strategy amongst flora and fauna. PO activities of bivalve hemocytes and proPO within the haemolymph have received much attention [146, 147]. Conversely, studies focussing on Hb-derived PO activities are relatively unheard-of (discussed below).

## Inducible phenoloxidase and (pseudo)peroxidase activities of hemocyanins and hemoglobins

Both arthropod and mollusc Hcs can be converted into PO-like enzymes upon physical disruption of the structural motifs in and around the dicopper centres. It is most important to open the entrance to the active site, yet such invasive structural alterations will eventually destroy the PO activity. Either through proteolysis or interactions with endogenous cofactors, placeholder residues (usually with aliphatic or aromatic chemistry) occluding the active sites are dislodged, thus permitting phenolic compounds to be processed into melanin precursors [21, 152]. The POs play vital roles in invertebrate development and contribute to counter-measures targeted towards infectious agents, e.g. hemocyte encapsulation/nodulation and using toxic quinones to kill pathogens [153]. POs (tyrosinases [EC 1.14.18.] and catecholoxidases [EC 1.10.3.1]) and Hc-d POs catalyse the *ortho*-hydroxylation of monophenols (_l_-tyrosine) into diphenols and subsequently oxidise the *o*-diphenols (_L_-dihydroxphenylalanine) into quinones (dopachrome) [154–157]. Arthropod Hc-d PO can convert 5,6-dihydroxyindole directly into melanin, a very resistant polymer net which invaders cannot penetrate [158]. Not all Hcs or POs can carry out the hydroxylation step. Recent data imply that an asparagine residue and a glutamate residue located near the CuB binding site are essential for tyrosinase activity. These residues fix a conserved water molecule and lower its pK value to disrupt the hydrogen from the monophenols (i.e. deprotonate), so the resulting phenolate can bind to CuA to initiate the enzymatic cycle [159–161]. The proton will be bound by this water molecule to form a hydronium ion (H_3_O^+^). After release of the final product, namely o-quinone, a hydroxyl group bridges the two copper ions but will be discarded as a water molecule after obtaining a hydrogen back from the hydronium ion. Upon replacing either the asparagine or glutamine residues with different amino acids only catecholoxidase activity is possible [161]. In the absence of a true PO, chelicerates rely on the inducible PO activity of Hc as a substitute [28, 30, 162, 163, 203, 204]. Lately, Hc was found to be a major component of clots formed during hemostasis in the spider, *Acanthoscurria geniculata*. It is postulated that Hc, like PO, enables protein cross-linking and sclerotisation of the cuticle post-moulting [32]. These findings are supported by earlier studies where Hc was present in abundance throughout the cuticles of shrimp (*Penaeus japonicus*) and tarantula (*Eurypelma californicum*) exoskeletons [164, 165].

Both mono-dimeric (31.2 kDa) and hetero-tetrameric (~60 kDa) conformational states of blood clam (*S. kagoshimensis)* Hb were found to possess PO-like activity. In vitro, Hbs could oxidise diphenols (catechol and _*L*_-DOPA; Table 3) to quinones (dopachrome) but were unable to carry out the initial hydroxylation reaction on monophenols [145]. Catalytic turnover was enhanced ~20 % by the polar solvent isopropanol, although exposure to SDS and trypsin led to a 75 % reduction in activity. SDS and ionic liquids are known to induce transient activity in many enzymes, including POs and Hc-d POs [155, 166, 167]. Bivalve Hb may be particularly sensitive to SDS-driven denaturation and tryptic digestion. Thermal and pH ranges of Hb-d PO as well as kinetic parameters such as substrate binding efficiencies (*K*_m_) for catechol and l-DOPA are similar to arthropod and mollusc Hc-d POs, notably cuttlefish (*Sepia officinalis*), snails (*Helix pomatia*) and crabs (*Charybdis japonicus*) (Table 3) [144, 145, 168–170]. Hb-d PO activity can be inhibited by known tyrosinase inhibitors (e.g.1-phenyl, 2-thiourea), standard metal chelators (EDTA, DETC) and antioxidants (ascorbic acid) in a similar way to POs [145]. The question remains, however, is Hb a latent PO or simply able to oxidise phenols non-specifically due to the presence of a transient metal ion within the heme cofactor?

**Table 3:** Inducible *o*-diphenoloxidase activity in hemoglobin versus hemocyanin

	Substrate kinetics			References
	Catechol	Dopamine	l-Dopa	
**Hemoglobins**				
*Scapharca kagoshimensis*	HbI—*K*_m_ = 5.7 mM HbII—*K*_m_ = 2.71 mM	–	HbI—*K*_m_ = 2.0 mM HbII—*K*_m_ = 1.22 mM	[145]
*Tegillarca granosa*	*K*_m_ = 0.097 mM	–	*K*_m_ = 1.44 mM	[144]
**Hemocyanins**				
*Charybdis japonica* (A)	–	–	*K*_m_ = 2.9 mM	[172]
*Eurypelma californicum* (A)	*K*_cat_/*K*_m_ = 0.20 ± 0.03 mM^.^s^−1^	*V*_max_ = 5.5 ± 0.4 µM.s^−1^ *K*_m_ = 1.45 ± 0.16 mM *K*_cat_/*K*_m_ = 3.91 ± 0.55 mM^.^s^−1^	*K*_cat_/*K*_m_ = 0.59 ± 0.08 mM^.^s^−1^	[168]
*Helix pomatia* (M)	*K*_m_ = 2.6 mM	–	–	[173]
*Helix vulgaris* (M)		*V*_max_ = 0.137 mM min^−1^ *K*_m_ = 2.86 mM	*V*_max_ = 0.018 mM min^−1^ *K*_m_ = 0.77 mM	[25]
*Limulus polyphemus* (A)	–	*V*_max_ = 4.7 ± 0.2 μmol min^−1^ *K*_m_ = 1.3 ± 0.1 mM	–	[170]
*Nephrops norvegicus* (A)	*V*_max_ = 5.84 ± 0.24 μmol min^−1^ *K*_m_ = 9.85 ± 0.89 mM [4-methylcatechol]	*V*_max_ = 2.4 ± 0.07 μmol min^−1^ *K*_m_ = 0.431 ± 0.04 mM	–	[174]
*Panulirus argus* (A)	*V*_max_ = 0.161 ± 0.005 ΔAbs min^−1^ *K*_m_ = 7.174 ± 0.487 mM	*V*_max_ = 0.143 ± 0.003 ΔAbs min^−1^ *K*_m_ = 0.181 ± 0.001 mM	*V*_max_ = 0.112 ± 0.002 ΔAbs min^−1^ *K*_m_ = 2.565 ± 0.115 mM	[175]
*Rapana venosa* FU-a (M)	–	*K*_m_ = 6.53 mM	*K*_m_ = 2.0 mM	[176]
*Sepia officinalis* (M)	*K*_m_ = 4.2 mM	–	*K*_m_ = 2.4 mM	[173]

*A* arthropod, *M* mollusc

In 2007, a seminal paper published by Jiang and co-workers described the pseudoperoxidase activity of human metHb and the PO activity of horseshoe crab Hc in the presence of extracellular proteases released by bacteria and fungi [24]. Hb and Hc were ‘switched on’ upon binding to bacterial ligands, LPS (Gram−) and LTA (Gram+), but were unaffected by laminarin. The generation of superoxide anions (O_2_^−^) by metHb correlated positively with concentrations of microbial stimulants, and equally, was suppressed by the addition of superoxide dismutase. Furthermore, increases in metHb ROS production were recorded inside erythrocytes exposed to different strains of *S. aureus*, highlighting the significance of this activity in vivo [24]. The peroxidase potential of Hb appears to be conserved amongst metazoans. Clam Hb can oxidise guaiacol (a methylated derivative of catechol) in the presence of H_2_O_2_ [144]. The catalytic turnover of phenols into quinones, whether it is by Hc, PO or Hb, generates volatile by-products [21, 24, 144, 145]. These oxidase-related enzymatic by-products boast significant broad-spectrum antimicrobial properties [171] evidenced by mollusc Hb’s inability to kill microbes in the presence of the ion scavenger, glutathione [144]. ROS formation by OTPs arises independently of immune signalling cascades and, therefore, provides an instantaneous assault on pathogens.

## Hemocyanin-derived cryptides

Hcs are acute phase proteins contributing to host recognition of non-self, pathogen opsonisation and agglutination, hemolysis, melanin biogenesis and virustasis, all of which have been reviewed in detail by Coates and Nairn (2014) [21]. The following section, however, is concerned primarily with the encrypted AMPs of Hc.

**Fig. 5:**
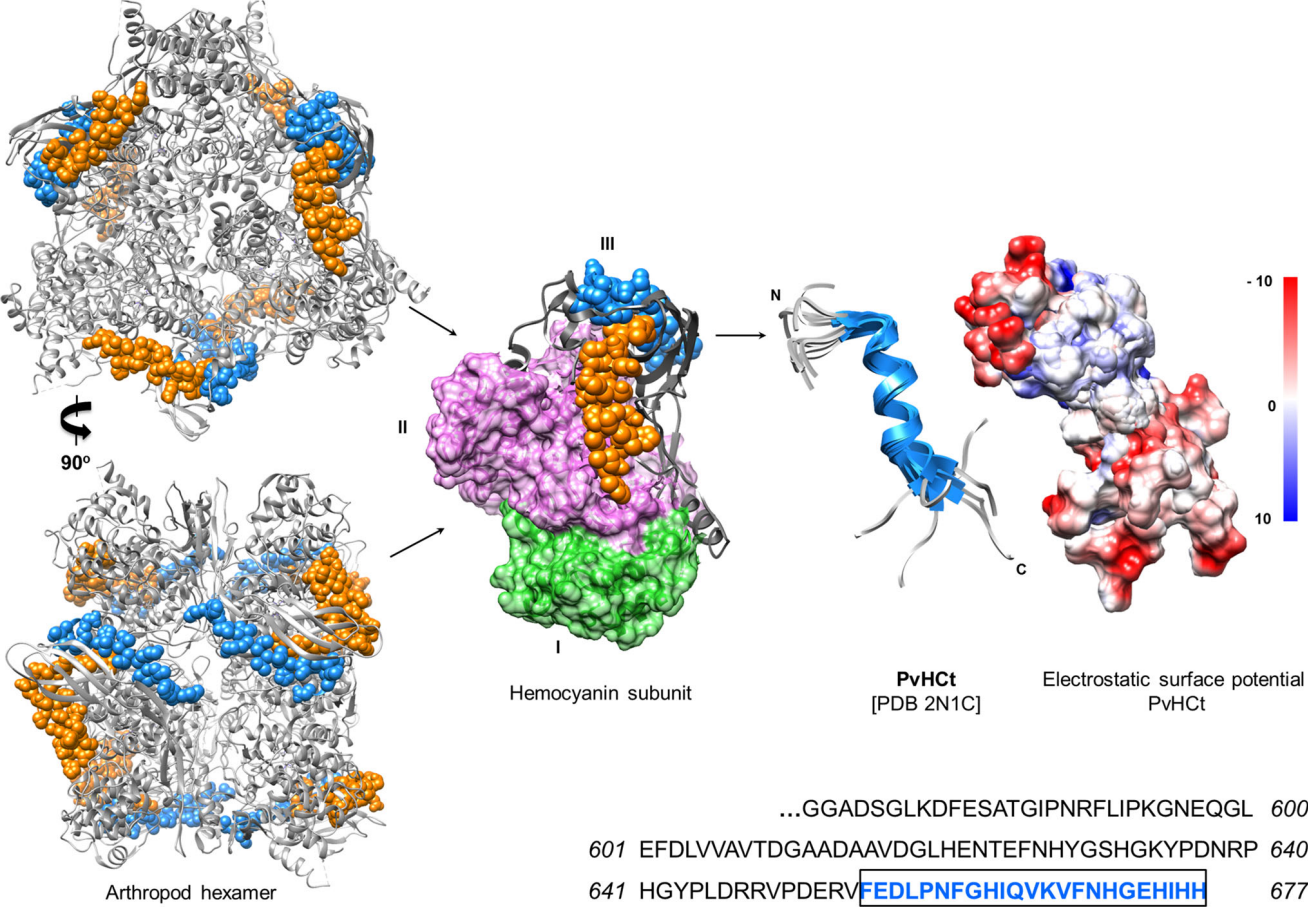
Arthropod hemocyanin-derived antimicrobial peptides. The crystal structure of *Panulirus interruptus* hemocyanin (PDB 1HC1) is used to illustrate the location of the encrypted peptides: PvHCt (FEDLPNFGHIQVKVFNHGEHIHH: *blue*), astacidin 1 (FKVQNQHGQVVKIFHH: *blue*) from crayfish, and PsHCt2 (LVVAVTDGDADSAVPNLHENTEYNHYGSHGVY: *orange*) from shrimp on the hemocyanin hexamer (~420 kDa) and corresponding subunit (~70 kDa). Hemocyanin subunit domains I and II are *coloured green* and *purple*, respectively. Both peptides are located on the C-terminal subunit (III) of the hemocyanin where they can be liberated through proteolysis. The shrimp peptide (PvHCt) is linear, α-helical and amphipathic, with an overall net-negative charge (theoretical *pI* = 6.1) as revealed by NMR. The electrostatic surface potential was calculated using UCSF Chimera [202]. PvHCt was isolated from *Litopenaeus vannamei* hemolymph and displays strict fungicidal activity. Both peptides are exposed to the environment even in the hexameric aggregation state. See also [21, 183]

Hc-derived peptides were first isolated from hemolymph plasma of commercially relevant shellfish species, one from *Litopenaeus vannamei* (PvHCt) and two from *Penaeus stylirostris* (Table 4) [177]. PvHCt failed to inhibit the growth of 17 bacterial species (both Gram +/−), yet revealed its exclusive antifungal activity at concentrations ranging from 3 to 50 µM (Table 4). Most recently, the structure of PvHCt was solved using a combination of ^1^H NMR and circular dichroism (Fig. 5). PvHCt is present in an unordered state in solution, and when incorporated into zwitterionic (DPC) micelles this histidine-rich peptide folds into a linear, amphipathic, α-helical structure with an overall net-negative charge (*pI* = 6.16) (Fig. 5) [178]. Amphipathicity is a key feature of most pore-forming AMPs. Hyphae and spores of *F. oxysporum* were damaged irreversibly within 90 min of PvHCt (20 µM) treatment due to its gross accumulation on the exoplasmic side of the fungal cell wall, but were not dependent on interactions with ergosterol. Cellular pathologies included ‘leakiness’ (4 kDa flux), plasma membrane deterioration, fewer lipid bodies and effete mitochondria. A cationic peptide, termed FCHc-C2, was manufactured recombinantly from the cDNA of shrimp (*Fenneropenaeus chinensis*) Hc [179]. FCHc-C2 and PvHCt share high sequence homology >90 %, the only differences being an aspartate is substituted with valine and a glycine is substituted with lysine (Table 4). Amphipathicity does not appear to differ significantly between these two peptides (Supp. Figure 2), yet unlike PvHCt, FCHc-C2 was active against Gram +/− bacteria as well as fungi. The (conventional) basic charge of FCHc-C2 (aided by an additional histidine on the hydrophilic side of the helix) might permit non-selective electrostatic interactions with a broader range of microbes.

**Table 4:** Anti-infective activity of hemocyanin-derived cryptides, and hemerythrin

Organism	Conformational state	Size	Charge	Activity (MIC or LD_50_)*	References
**Hemerythrin**					
Medicinal Leech (*Hirudo medicinalis*)	Octamer Up-regulation of Hr confirmed via 2D SDS-PAGE, protein accumulation occurred in tissues of the CNS	~108 kDa		**Antibacterial:** Gram+/− *Escherichia coli and Micrococcus luteus*	[189]
Rag (sand) worm (*Hediste diversicolor*)	MPII, 119 amino acids	13.7 kDa		**Antibacterial:** Gram+/− *Kocuria kristinae, Micrococcus luteus, Escherichia coli and Vibrio alginolyticus*	[192]
**Hemocyanin**					
Abalone (*Haliotis tuberculata*)	YKKFGYRYDSLELEGRSISRIDELIQQRQEKDRTFAGFLLKGF (linear α-helix, termed haliotisin)	5.2 kDa	Cationic *pI* = 9.66	**Antibacterial:** Gram+/− *Bacillus subtilis* (0.3–3 µM) *Erwinia carotovora* (0.8–2.6 µM)	[187]
Crayfish (*Pacifastacus leniusculus*)	FKVQNQHGQVVKIFHH (termed astacidin 1) (β-sheet at pH 4)	1.9 kDa	Cationic *pI* = 10.6	**Antibacterial:** Gram+/− *Bacillus megaterium* (1.9 µM) *Bacillus subtilis* (15 µM) *Staphylococcus aureus* (>20 µM) *Micrococcus luteus* (12.8 µM) *Pseudomonas aeruginosa* (>20 µM) *Escherichia coli* (15 µM) *Proteus vulgaris* (>20 µM) *Shigella flexneri* (15 µM) **Antifungal:** *Candida albicans* (6.3 µM) *Trichosporon beigelii* (6.3 µM) *Malassezia furfur* (12.5 µM) *Trichophyton rubrum* (25 µM)	[180, 182]
Shrimp (*Fenneropenaeus chinensis*)	FEVLPNFKHIQVKVFNHGEHIHHH (termed FCHc-C2) LVVAVTDGEADAAVEGLHDNTDFIHYGSHGKYPDNRPHGYPLD	4.9 kDa ~8 kDa	Cationic *pI* = *7.98* Anionic *pI* = 4.4	**Antibacterial:** Gram+/− *Micrococcus luteus* (1.3–13 µM) *Aeromonas hydrophila* (10.6–26 µM) *Pseudomonas aeruginosa* (13–26 µM) *Vibrio anguillarum* (13–26 µM) **Antifungal:** *Botrytis cinerea* (1.3–21.2 µM) *Colletotrichum orbiculare* (1.1–2.6 µM) *Fusarium oxysporum* (10.6–26 µM) *Pestalotia diospyri* (>21.2 µM) *Pythium ultimum* (>21.2 µM) *Sclerotinia sclerotiorum* (>21.2 µM)	[179]
Shrimp (*Litopenaeus vannamei*)	FEDLPNFGHIQVKVFNHGEHIHH (termed PvHCt) (PDB; 2N1C)	2.76 kDa	Anionic *pI* = 6.1	**Antifungal:** *Alternaria brassicola* (3.2–6.3 µM) *Fusarium culmorum* (6.3–12.5 µM) *Fusarium oxysporum* (6.3–12.5 µM) *Nectria hematococca* (3.2–6.3 µM) *Neurospora crassa* (25–50 µM) *Tricoderma viridae* (3.2–6.3 µM)	[177, 178]
Shrimp (*Penaeus stylirostris*)	LVVAVTDGDADSAVPNLHENTEYNHYGSHGVY VTDGDADSAVPNLHENTEYNHYGSHGVYPDK	8.3 kDa 7.9 kDa	Anionic *pI* = 4.22 Anionic *pI* = 4.32	**Antifungal:** *Fusarium oxysporum*	[177]
Snail (*Helix aspersa*)	Functional Unit-H	~60 kDa		**Antibacterial:** Gram+/− *Staphylococcus aureus* *Streptococcus epidermidis* *Escherichia coli*	[186]
Spider (*Acanthoscurria rondoniae*)	IIIQYEGHKH (termed rondonin)	1.2 kDa	Cationic *pI* = 7.9	**Antifungal:** Beauveria bassiana (1.1 µM) *Candida albicans* (8.4–16.8 µM) *Candida krusei* (16.8 µM) *Candida glabrata* (8.4 µM) *Candida parapsilosis* (16.8 µM) *Candida tropicalis* (8.8 µM) *Candida guilliermondii* (16.8 µM)	[181]
Whelk (*Rapana venosa*)	Functional Units B and E ELVRKNVDHLSTPDVLELV 94 % coverage and 72 % identity with *Helix lucorum* Hc 73 % coverage and 70 % identity with *Rapana venosa* Hc2 FU-e;	~50 kDa 2.2 kDa	Anionic *pI* = 4.54	**Antibacterial:** Gram + *Staphylococcus aureus*	[185]

* Minimum inhibitory concentration (MIC) or LD_50_ values were not available for all (poly) peptides listed

Other Hc-derived peptides, namely astacidin 1 and rondonin, were recovered from the hemolymph of immune-stimulated crayfish (*Pacifastacus leniusculus* [180]) and spiders (*Acanthoscurria rondoniae* [181]), respectively. In solution, astacidin1 forms a β-sheet structure and is active against many bacteria [180] and fungi [182] (Table 4). These peptides are located on the surface of the Hcs and, therefore, exposed to the environment (Fig. 5 [21, 183]). Characterisations of truncated astacidin 1 variants revealed a dependency on the N-terminal residues (FKVQNQHGQVVKIFHH-*COOH*) for effective microbe killing. Similar to PvHCt, astacidin 1 functions optimally at acidic pH and causes injury to the external membranes of fungi, creating trans-bilayer pores with radii ~2 nm. Rondonin, PvHCt and astacidin 1 all originated from the C-terminal domains (III) of their precursor Hcs, a structurally conserved region organised into a seven-stranded anti-parallel β-barrel (Figs. 1 and 5). Each peptide is likely detached from Hc via directed proteolysis. Aspartyl (pepstatin) and cysteine (E-64) protease inhibitors impeded the production of astacidin 1 in crayfish, indicating the peptide may be cleaved from Hc by cysteine-like proteases released by lysosomes [180]. Concentrations of Hc-derived AMPs in the hemolymph of shrimp and crayfish increased significantly in the presence of microbial ligands, LPS and β-glucan [177, 180]. Therefore, Hc circulating freely in the haemolymph is an immediate source of immune mediators.

Although diverse antimicrobial and antiviral properties of mollusc Hc oligomers and several FUs have been noted [21, 184–186], mollusc Hc-derived peptides have received little attention. An interrogation of whelk (*Rapana venosa*) hemolymph led to the identification of a peptide with sequence similarities to a conserved motif at the N-terminus of many Hc FUs [185]. This whelk Hc-derived peptide did not inhibit the growth of *S. aureus* or *Klebsiella pneumoniae*; therefore, its physiological function is a mystery. An in silico study performed on abalone (*Haliotis tuberculata*) Hc indicated the region between the α-helical and β-sandwich domains of FU-e contained encrypted AMPs [187]. A number of synthetic polypeptides resembling this region were antagonistic towards *Erwinia carotovora* and *B. subtilis* in vitro (Table 4). Membrane perturbations visible in electron micrographs of Hc-treated bacteria suggested the amphipathic peptide may act as a pore former. Predictive modelling of the strongest bioactive peptide (termed haliotisin; Table 4) revealed a linear, α-helical structural conformation. Moreover, the peptide is positioned at the surface of the Hc protomer and is flanked by a series of trypsin and chymotrypsin cleavage sites, making it highly accessible during sepsis [187].

So far, no cysteine residues or disulphide bridges were found within any known Hc-derived AMP. This may be advantageous since the peptides can bind more easily to pathogens and be transported more readily through a membrane into the interior, rather than a stiff peptide. Each peptide contains at least three (up to eight) positively charged residues (H, R, K) and differ substantially in their net electrical charges, *pI* = 4–11 (Table 4; Fig. 5). The mode of action of Hc-derived AMPs may not be restricted to pore formation, as evidence discussed here hints to a possible role interfering with subcellular organelles.

## Hemerythrin and innate immunity

Hr functionality is poorly characterised when compared to Hb and Hc. Nevertheless, members of the Hr gene family, including myoHr, participate in respiration, heavy metal detoxification and aspects of innate immunity [188]. Responding to septic shock caused by *E. coli* and *Micrococcus luteus*, Hr expression increased significantly in the leech, *Hirudo medicinalis* [189]. Using a 2-DIGE approach, newly synthesised Hr accumulated within tissues of the central nervous system, referred to as neuro-hemerythrin. Hr expression was also spatially distributed in peripheral tissues such as muscle, the walls of blood vessels and nephridia (an excretory organ analogous to vertebrate kidneys). The dual functionality of Hr in leech immunity was contemplated; provision of oxygen to fuel the metabolic costs of immune activity, and the sequestration of iron needed by microbes to grow [189].

Metalloprotein II (MPII) is an antibacterial protein found in the coelomic fluid of *Hediste* (*Nereis*) *diversicolor* and other polychaetes [190–192]. It is a cadmium binding protein related to the Hr family, ~81 % similar to myoHr [193, 194], and can be produced within specialised coelomocytes (granulocytes type I), somatic muscle cells and the lining of the gut [188, 195]. MPII and myoHr are monomeric isoforms of Hr subunits, displaying almost identical structural architecture (four α-helix bundle). Upon immune stimulation with intact microbes (*Vibrio alginolyticus, E. coli and M. luteus*) or endotoxins, MPII is expelled into the coelomic fluid by granulocytes type I [192]. Concurrently, the enzyme PO is released by granulocytes type II. MPII likely provides the oxygen needed to catalyse phenol hydroxylation/oxidation, thereby facilitating the eventual biogenesis of melanin. This is quite interesting as Hc and Hb are also involved in converting phenols into (semi)quinone derivatives (see previous sections). The antibacterial properties of MPII are disrupted in the presence of iron or when the coelomic fluid is pre-treated with specific antibodies raised against MPII [192]. Certain sipunculids contain differential coelomocytes (pink blood cells) that express variants of Hr [5, 196]. Depending on their location within the body, cell-specific Hrs bind oxygen with varying affinities: low, moderate and high. Coelomocyte hematopoiesis following severe blood loss (exsanguination) in the peanut worm, *Phascolosoma esculenta*, accompanies the *de novo* synthesis of Hr [53]. Isoforms of Hr and myoHr have also been found in the salivary complex of the hematophagous leech, *Haementeria depressa,* and during anterior tissue regeneration of the earthworm, *Perionyx excavatus* [197, 198]. These data signify Hr is a multi-functional protein extending beyond its traditional role as a vehicle for molecular oxygen.

In 2011, a 10-kDa polypeptide was extracted from the exoskeleton of a baculovirus-infected crustacean, *Pleuroncodes planipes* [199]. Protein extracts were capable of inhibiting up to 99.5 % of polyhedrosis nuclear virus replication. An acidic region of this polypeptide, VFYANLDEEHK, shared 100 % coverage and 91–100 % amino acid sequence identity with Hr-like protein subunits from annelids, *Scoloplos armiger* (Accession no. XP_013415662) and *H. medicinalis* (Accession no. Q674M7), and a brachiopod, *Lingula anatina* (Accession no. CAP08294). The authors compared their polypeptide to myoHr, yet in the absence of a known Hr within the Crustacea, caution and further information are required before categorising this protein as an immune effector.

The use of Hr as a defence strategy is not only employed by metazoan hosts. The aquatic pathogen, *Aeromonas hydrophila*, produces a single-domain Hr when inside the cytosol of Japanese eel macrophages to sense O_2_ and detoxify ROS [200]. Differential expression of Hr under extreme O_2_ conditions (hypoxic, hyperoxic) was recorded in wild-type *A. hydrophila.* The bacterium was also able to cope with high-level exposure to H_2_O_2_. Disruption of the Hr gene in *A. hydrophila* mutants (M85) led to a 77 % reduction in survival when incubated with macrophages. Virulence of *A. hydrophila* is dependent on its ability to escape phagosomes using flagellar movements [201]. No differences in motility were found between the wild-type and Hr mutants; therefore, Hr is an important factor in *A. hydrophila* pathogenesis.

## Further considerations

In many vertebrate and invertebrate systems, the presence of pathogens and parasites in the blood/hemolymph can lead to the de novo synthesis of OTPs (Hb, Hc and Hr). Extracellular proteases that are secreted by microbes target Hb and Hc, leading to the production of ROS/RNS, the conversion of phenolic substrates and release of encrypted immune peptides. The activities of OTP cryptides do not depend on their respective metal prosthetic groups. Hb in the absence of the heme cofactor retains its antimicrobial potency, and likewise, all Hc-derived AMPs originate from the C-terminal domains of arthropod Hc subunits and mollusc Hc functional units where they are not influenced by the distant dicopper centres located within the α-helical structural domain.

For each OTP there are many bona fide enzymes employing heme, di-iron or dicopper catalytic units: Hb—peroxidase and cytochrome P450; Hc—tyrosinase and ascorbate oxidase; Hr—ribonucleotide reductase and methane mono-oxygenase [4]. Therefore, is enzymatic activity or altered functionality of Hb/Hc/Hr a coincidence of subtle structural rearrangements or interchangeable roles in respiration, detoxification and immunity? A link between OTPs and immunity may be the mitigation or remediation of damage incurred by the host when mounting an immune response. RNS and ROS are equally harmful to the host as they are to microbes, highlighting the essential need for their production to be tightly regulated.

The evolution of OTPs is likely influenced by two co-evolving systems: (1) optimising the precise delivery of dioxygen and storing it within cells (i.e. respiration), and (2) serving as an immediate and efficacious anti-infective agent (i.e. immunity).

## Electronic supplementary material

Below is the link to the electronic supplementary material.

Supplementary material 1 (DOCX 1697 kb)

## Abbreviations

AMP: Antimicrobial peptide
2-DIGE: 2-Dimensional gel electrophoresis
GE-Hb: Giant extracellular hemoglobin
Hp: Haptoglobin
Hb: Hemoglobin
Hc: Hemocyanin
Hr: Hemerythrin
IFN: Interferon
LPS: Lipopolysaccharide
LTA: Lipoteichoic acid
metHb: Methemoglobin
Mb: Myoglobin
PRR: Pathogen recognition receptor
PAMP: Pathogen associated molecular pattern
PGN: Peptidoglycan
PO: Phenoloxidase
RBCs: Red blood cells
RNS: Reactive nitrogen species
ROS: Reactive oxygen species
SDS: Sodium dodecyl sulphate
SNPs: Single nucleotide polymorphisms
TLR: Toll-like receptor
WBCs: White blood cells

## Footnotes

1 Hemolymph and coelomic fluid are the invertebrate equivalents to blood.
