# Supplementary material for: Immunological properties of oxygen-transport proteins: hemoglobin, hemocyanin and hemerythrin
Source: Cell Mol Life Sci. 2016 Aug 12;74(2):293–317. doi: 10.1007/s00018-016-2326-7 (PMC5219038; doi:10.1007/s00018-016-2326-7)
Supplement: Supplementary file 1 — Supplementary material 1 (DOCX 1697 kb) [file 18_2016_2326_MOESM1_ESM.docx]

**Supplementary Table 1** Extended list of hemoglobin (and myoglobin) derived antimicrobial peptides

|  | **Sequence** | **Source** | **Charge** | **Reference** |
| --- | --- | --- | --- | --- |
| **Bovine** |  |  |  |  |
| α (33 – 61) | FLSFPTTKTYFPHFDLSHGSAQVKGHGAK | Found in the gut of the cattle tick,  *Rhipicephalus microplus***^1^** | +5 | Fogaca et al. (1999) |
| α (33 – 61)-*amidated* | FLSFPTTKTYFPHFDLSHGSAQVKGHGAK-*NH_2_* |  | +5 | Machado et al. (2007)  Carvalho et al. (2015)  Belmonte et al. (2012) |
| α (40 – 61)-*amidated* | KTYFPHFDLSHGSAQVKGHGAK-*NH_2_* |  | +5 |  |
| α (98 – 114) | FKLLSHSLLVTLASHLP |  | +3 |  |
| α (1 – 23) | VLSAADKGNVKAAWGKVGGHAAE | Peptic digestion of hemoglobin, *in vitro* | +2 | Froidevaux et al. (2001) |
| α (107 – 136) | VTLASHLPSDFTPAVHASLDKFLANVSTVL | **24 hemocidins**  **6 hemocidins** | +1 | Daoud et al. (2005)  Nedjar-Arroume et al. (2006) |
| α (107–141) | VTLASHLPSDFTPAVHASLDKFLANVSTVLTSKYR |  | +3 |  |
| α (137 – 141) | TSKYR (Neokyotorphine) |  | +2 |  |
| α (133 –141) | STVLTSKYR |  | +2 |  |
| β (126 – 145) | QADFQKVVAGVANALAHRYH |  | +3 |  |
| **α - chain:**  1-23,27, 28, 29, 32; 33-45,46,66, 83, 97, 98; 34-46, 66, 83, 98; 36-45,97; 37-46, 98; 107-133, 136, 141; 133-141; 137-141 | VLSAADKGNVKAAWGKVGGHAAEYGAEALERMFLSFPTTKTYFPHFDLSHGSAQVKGHGAKVAAALTKAVEHLDDLPGALSELSDLHAHKLRVDPVNFKLLSHSLLVTLASHLPSDFTPAVHASLDKFLANVSTVLTSKYR |  |  | Nedjar-Arroume et al. (2008)**^2^** |
| **β - chain:**  1-13; 1-30; 114-145; 121-145; 126-145; 140-145 | MLTAEEKAAVTAFWGKVKVDEVGGEALGRLLVVYPWTQRFFESFGDLSTADAVMNNPKVKAHGKKVLDSFSNGMKHLDDLKGTFAALSELHCDKLHVDPENFKLLGNVLVVVLARNFGKEFTPVLQADFQKVVAGVANALAHRYH |  |  |  |
| **α (96 – 113)** *(or* ***P3****)* | **VNFKLLSHSLLVTLASHL**  KLLSHSLLVTLASHL  KLL**R**H**R**LLVTLASHL  V**R**FKLLSHSLLVTLASHL  **RR**FKLLSHSLLVTLASHL | Extracted directly from bovine erythrocytes | +3  +3  +5  +4  +5 | Zhang et al. (2015)  Hu et al. (2011) |
| **Crocodile** |  |  |  |  |
| α (1 – 19) | VLSSDDKCNVKAVWCKVAG | Hemoglobin was extracted from red blood cells and digested with pepsin | +1 | Srihongthong et al. (2012) |
| α (1 – 13) | VLSSDDKCNVKAV |  | 0 |  |
| α (16 – 26) | KVAGHLEEYGA |  | 0 |  |
| β (15 – 22) | WHKVDVAH |  | +2 |  |
| α (67 – 72) | HEAVNH |  | +1 |  |
| β (69 – 80) | ASFGEAVKHLDS |  | 0 |  |
| β (69 – 82) | ASFGEAVKHLDSIR |  | +1 |  |
| α (107 – 121) | VVVAIHHPGSLTPEV |  | +1 |  |
| α (107 – 124) | VVVAIHHPGSLTPEVHAS |  | +2 |  |
| α (107 – 128) | VVVAIHHPGSLTPEVHASLDKF |  | +2 |  |
| α (110 – 123) | AIHHPGSLTPEVHA |  | +2 |  |
| α (115 – 124) | AIHHPGSLTPEVHASLDKFL |  | +2 |  |
| α (A115-L124) | AAHYPKDFGL |  | +1 |  |
|  |  |  |  |  |
| **Human** |  |  |  |  |
| **I** α (1 – 32) | VLSPADKTNVKAAWGKVGAHAGEYGAEALERM | Present in serum, and cyanogen bromide treatment of purified hemoglobin | +1 | Mak et al. (2000)  Parish et al. (2001) |
| **II** α (33 – 76) | FLSFPTTKTYFPHFDLSHGSAQVKGHGKKVADALTNAVAHVDDM |  | +4 |  |
| **III** α (1 – 76) | VLSPADKTNVKAAWGKVGAHAGEYGAEALERMFLSFPTTKTYFPHFDLSHGSAQVKGHGKKVADALTNAVAHVDDM |  | (+5) |  |
| **IV** α (77 – 141) | PNALSALSDLHAHKLRVDPVNFKLLSHCLLVTLAAHLPAEFTPAVHASLDKFLASVSTVLTSKYR |  | (+7) |  |
| **V** β (56 – 146) | GNPKVKAHGKKVLGAFSDGLAHLDNLKGTFATLSELHCDKLHVDPENFRLLGNVLVCVLAHHFGKEFTPPVQAAYQKVVAGVANALAHKYH |  | (+11) |  |
| **VI** β (1 – 55) | VHLTPEEKSAVTALWGKVNVDEVGGEALGRLLVVYPWTQRFFESFGDLSTPDAVM |  | 0 |  |
| β (116 – 146) | HHFGKEFTPPVQAAYQKVVAGVANALAHKYH |  | +6 |  |
| β (56 – 72) | GNPKVKAHGKKVLGAFS |  | +5 |  |
|  |  |  |  |  |
| hHEM-β | VCVLAHHFGKEFTPPVQAAYQKVVAGVANALAHKYH | Present in erythrocytes and in placental blood | +6 | Liepke et al. (2003) |
| Foetal Hb-γ | WQKMVTAVASALSSRYH |  | +3 |  |
|  |  |  |  |  |
| α (35 – 56) | SFPTTKTYFPHFDLSHGSAQVK | Menstrual secretions | +3 | Mak et al. (2004, 2007)**^2^** |
| β (115 –146) | AHHFGKEFTPPVQAAYQKVVAGVANALAHKYH |  | +6 |  |
|  |  |  |  |  |
| α 1-29 (12-29): | VLSPADKTNVKAAWGKVGAHAGEYGAEAL | Generated by a series of aspartic peptidases secreted by *Candida albicans* | +1 | Bochenska et al. (2013)**^2^** |
| β 1-21 (9-21); | VHLTPEEKSAVTALWGKVNVD |  | 0 |  |
|  |  |  |  |  |
| α (32 – 41) | FLSFPTTKTY | Endometrium | +1 | Deng et al. (2009) |
|  |  |  |  |  |
|  |  |  |  |  |
| **Whale myoglobin** |  |  |  |  |
| 1 – 55 | VLSEGEWQLVLHVWAKVEADVAGHGQDILIRLFKSHPETLEKFDRFKHLKTEAEM | Cyanogen bromide treatment of purified myoglobin | +1 | Mak et al. (2000) |
| 56 – 131 | KASEDLKKHGVTVLTALGAILKKKGHHEAELKPLAQSHATKHKIPIKYLEFISEAIIHVLHSRHPGDFGADAQGAM |  | (+11) |  |
| 132 – 153 | NKALELFRKDIAAKYKELGYQG |  | +2 |  |

^1^Formerly *Boophilus microplus*

^2^More peptides (hemocidins) are listed in the original manuscript

**References**

- Carvalho, L.A.C., Remuzgo, C., Perez, K.R., Machini, M.T., 2015. Hb40-61a: Novel analogues help expanding the knowledge on chemistry, properties and candidiacidal action of this bovine α-hemoglobin-derived peptide. Biochem. Biophys. Acta 1848, 3140-3149.
- Belmonte, R., Cruz, C.E., Pires, J.R., Daffre, S., 2012. Purification and characterization of Hb 98-114: A novel hemoglobin-derived antimicrobial peptide from the midgut of *Rhipicephalus* (*Boophilus*) *microplus*. Peptides 37, 120-127.
- Bochenska, O., Rapala-Kozik, M., Wolak, N., et al., 2013. Secreted aspartic peptidases of *Candida albicans* liberate bactericidal hemocidins from human hemoglobin. Peptides 48, 49-58.
- Daoud, R., Dubois, V., Bors-Dodita, L., Nedjar-Arroune, N., Krier, F., Chihib, N-E., Mary, P., Kouach, M., Briand, G., Guillochon, F., 2005. New antibacterial peptide derived from bovine hemoglobin. Peptides 26, 713-719.
- Deng, L., Pan, X., Wang, Y., Wang, L., Zhou, X.E., Li, M., Feng, Y., Wu, Q., Wang, B., Huang, N., 2009. Hemoglobin and its derived peptides may play a role in the antibacterial mechanism of the vagina. Human Repro. 24, 211-218.
- Fogaca, A.C., da Silva, P.I. et al., 1999. Antimicrobial activity of a bovine hemoglobin fragment in the Tick *Boophilus microplus*. J. Biol. Chem. 274, 25330-25334.
- Froidevaux, R., Krier, F., Nedjar-Arroume, N., et al., 2001. Antibacterial activity of a pepsin derived bovine hemoglobin fragment. FEBS Letts. 491, 159-163.
- Hu, J., Xu, M., Hang, B., Wang, L., Wang, Q., Chen, J., Song, T., Fu, D., Wang, Z., Wang, S., Liu, X., 2011. Isolation and characterization of an antimicrobial peptide from bovine hemoglobin α-subunit. World J. Microbiol. Biotechnol. 27, 767-771.
- Liepke, C., Baxmann, S., Heine, C., et al., 2003. Human hemoglobin-derived peptides exhibit antimicrobial activity: a class of host defense peptides.
- Machado, A., Sforca, M.L., Miranda, A., Daffre, S., Pertinhez, T.A., Spisni, A., Miranda, M.T.M., 2007. Truncation of the amidated fragment 33-61 of bovine α-hemoglobin: effects on the structure and anticandidial activity. Biopolymers 88, 413-426.
- Mak, P., Wojcik, K., Silberring, J., Dubin, A., 2000. Antimicrobial peptides derived from heme-containing proteins: Hemocidins. Antonie von Leeuwenhoek 77, 197-207.
- Mak, P., Siwek, M., Pohl, J., Dubin, A., 2007. Menstrual hemocidins Hb115-146 is an acidophilic antibacterial peptide potentiating the activity of human defensins, cathelicidin and lysozyme. Am. J. Reprod. Immunol. 57, 81-91.
- Mak, P., Wojcik, K., Wicherek, L., Suder, P., Dubin, A., 2004. Antibacterial peptides in human menstrual blood. Peptides 25, 1839-1847.
- Nedjar-Arroume, N., Dubois-Delva, V., Adje, E.Y., Traisnel, J., Krier, F., Mary, P., Kouach, M., Briand, G., Guillochon, D., 2008. Bovine hemoglobin: An attractive source of antibacterial peptides. Peptides 29, 969-977.
- Nedjar-Arroume, N., Dubois-Delva, V., Miloudi, K., Daoud, R., Krier, F., Kouach, M., Briand, G., Guillochon, D., 2006. Isolation and characterization of four antibacterial peptides from bovine hemoglobin. Peptides 27, 2082-2089.
- Parish, C.A., Jiang, H., Tokiwa, Y., Berova, N., et al., 2001. Broad-spectrum antimicrobial activity of hemoglobin. Bioinorg. Med. Chem. 9, 377-382.
- Srihongthong, S., Pakdeesuwan, A., et al., 2012. Complete amino acid sequence of globin chains and biological activity of fragmented crocodile hemoglobin (*Crocodylus siamensis*). Protein J. 31, 466-476.
- Zhang, Q., Xu, Y., Wang, Q., Hang, B., Sun, Y., Wei, X., Hu, J., 2015. Potential of novel antimicrobial peptide P3 from bovine erythrocytes and its analogs to disrupt bacterial membranes *in vitro* and display activity against drug-resistant bacteria in a mouse model. Antimicrob. Agents Chemother. 59, 2835-2841


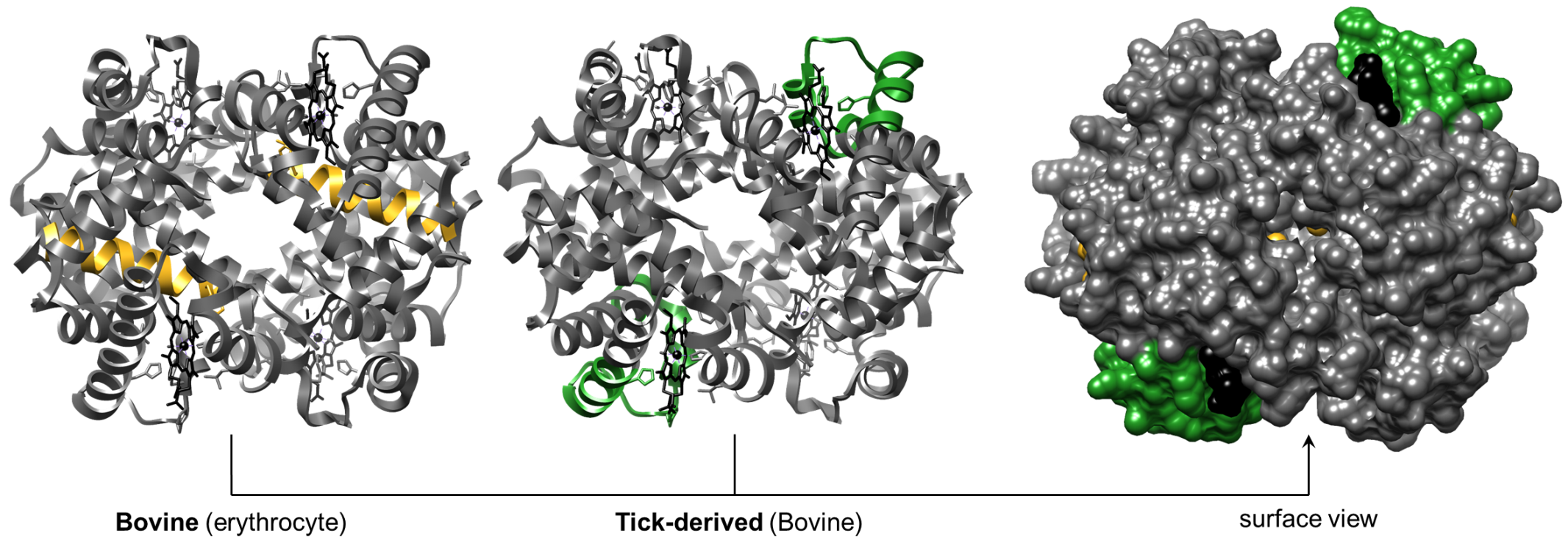


**Supplementary Figure 1**

Hemoglobin-derived peptides produced by bovine erythrocytes and by ticks (using bovine hemoglobin) are both represented on the surface view of the macromolecule (*Bos taurus*; PDB 2QSS). The erythrocyte hemocidins (yellow) appear buried within the hemoglobin tetramer, whereas, the tick-derived hemoglobin fragments (green) are easily accessible at the surface.

**
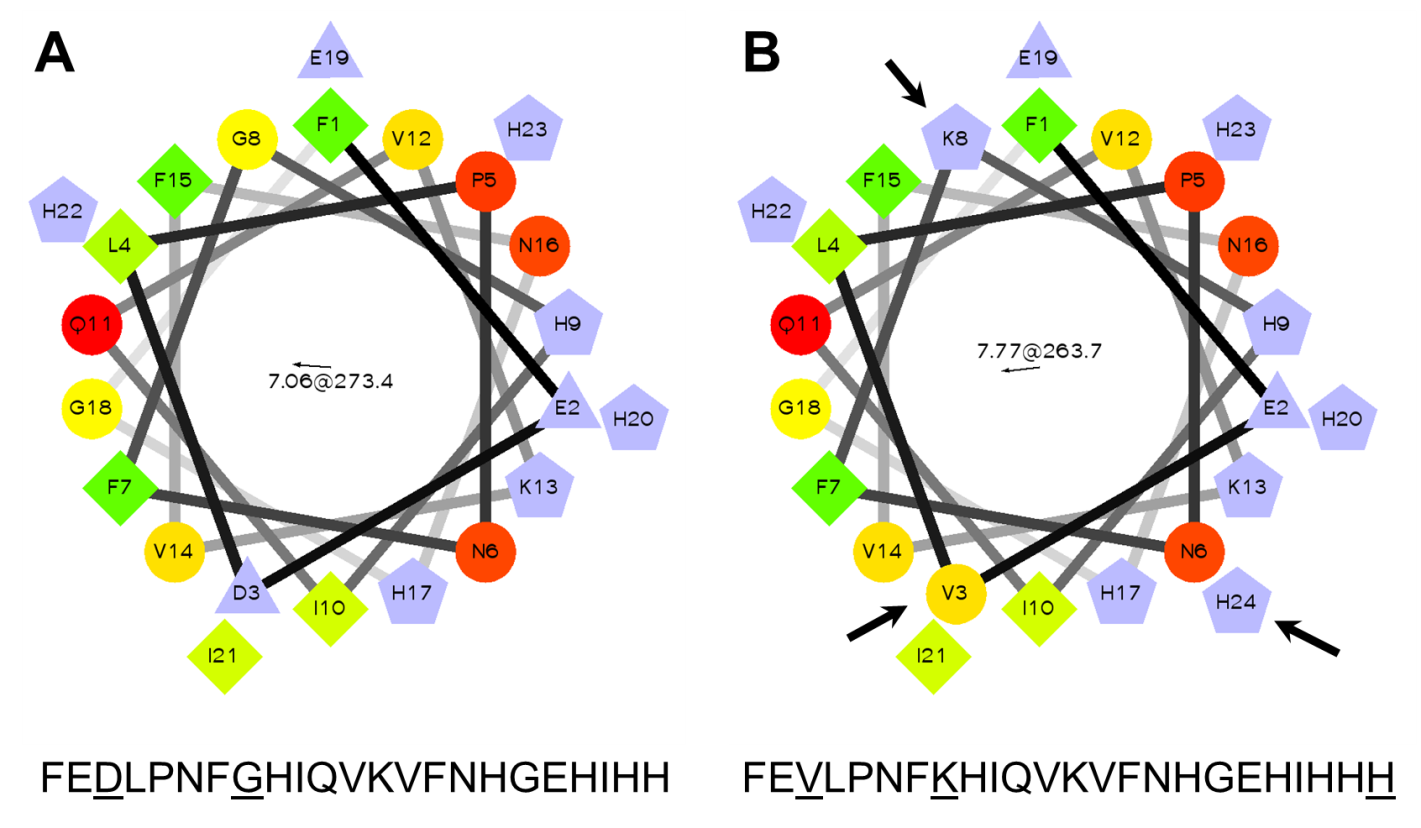
**

**Supplementary Figure 2**

Helical wheel representations of hemocyanin-derived cryptides from shrimp. A) PvHCt is an anionic, antifungal peptide from *Litopenaeus vannamei (pI = 6.1)* and B) FcHc-C2 is a cationic, broad-spectrum antimicrobial peptide from *Fenneropenaeus chinensis (pI = 7.98)*. The arrows indicate differences in the amino acid composition, and there is an additional histidine residue. Both peptides are amphipathic.

- Hydrophilic residues are represented as circles: hydrophilic residues are coded red with pure red being the most hydrophilic (uncharged) residue, and the amount of red decreasing proportionally to the hydrophilicity. The potentially charged residues are light blue
- Potentially negatively charged as triangles, and potentially positively charged as pentagons
- Hydrophobic residues are represented as diamonds: Hydrophobicity is color coded as well: the most hydrophobic residue is green, and the amount of green is decreasing proportionally to the hydrophobicity, with zero hydrophobicity coded as yellow.
